# Radiomics in spinal research: a narrative review

**DOI:** 10.3389/fbioe.2026.1769254

**Published:** 2026-09-11

**Authors:** Brian S. Tao, Katelyn A. Tao, Mario Keko, Nazim Haouchine, Ron Noah Alkalay

**Affiliations:** 1 Chobanian & Avedisian School of Medicine, Boston University, Boston, MA, United States; 2 Stony Brooke University, Stony Brook, NY, United States; 3 Department of Orthopedic Surgery, Beth Israel Deaconess Medical Center and Harvard Medical School, Boston, MA, United States; 4 Department of Radiology, Brigham and Women’s Hospital, Boston, MA, United States

**Keywords:** intervertebral disc disease, malignant vertebral fractures, malignant vertebral lesions, narrative review, osteoporotic spine fracture, radiomics, spine imaging, Machine Learning

## Abstract

Radiomics has emerged as a transformative tool in medical imaging, offering the ability to extract complex quantitative features that are generally indiscernible to the human eye. These radiomic features can be interrogated non-invasively on medical imaging. Importantly, in contrast to traditional diagnostic methods reliant on a single, often scalar, measure, radiomics features can form a high-dimensional data space from imaging data suitable for machine learning. Within the framework of artificial intelligence, radiomic features can be harnessed for differentiating healthy from pathological tissue, risk-stratifying patients for benign and malignant fractures, and clinical outcome measures. This review presents an introduction to the methodology underlying radiomics feature selection, reproducibility, feature analysis and model building, assessment of model performance, and open-source libraries for extracting radiomics features from imaging. The review then highlights the application of radiomics in osseous and cartilaginous spinal imaging for identifying osteoporosis and the prediction of fragility vertebral fractures, chronic low back pain and the assessment of intervertebral disc degeneration and herniation, cancer metastatic spine disease and the differentiation of benign vs. malignant lesions and the classification of benign vs. malignant vertebral fractures. Throughout this stage, we endeavor to demonstrate how radiomics can analyze imaging biomarkers to detect subtle structural changes in vertebral bone microarchitecture, assess tissue quality and early-stage fractures, and identify radiomic biomarkers of low back pain chronicity and intervertebral disc heterogeneity that signal degeneration and herniation risk. The review culminates with the presentation of the current limitations and future research directions, including opportunities for integration with multi-omics analysis, highlighting radiomics’ potential for enhanced diagnostic accuracy and more personalized patient care. The application of radiomics in spinal imaging offers a promising avenue for improving non-invasive image early detection, risk stratification, and personalized management of spinal pathology, paving the way for more effective interventions and improved patient outcomes.

## 1 Introduction

Radiomics, a relatively recent area of precision medicine, provides a quantitative approach to medical imaging through advanced mathematical analysis of imaging data (Gillies et al., 2016). First introduced by Lambin et al. (2012), the idea of “radiomics” is founded on the assumption that images of biological tissue and structures contain disease-specific information undetectable to the human eye and thus, not accessible through conventional visual analysis of the generated image (Gillies et al., 2016; Alderson and Summers, 2020). Several landmark studies, including Aerts et al. (2014), introduced the idea of “radiomics signatures” for noninvasive prognostic biomarkers, with Parmar et al. (2015) introducing the use of machine learning and radiomics. Since then, radiomics has played an important role in personalized precision medicine (PPM) (Miles, 2020) and has been applied to analyze magnetic resonance imaging (MRI), computed tomography (CT), positron emission tomography (PET), and ultrasound (US) (Mannil et al., 2018; Masokano et al., 2020; Qin et al., 2021; Reuze et al., 2018; Zhu et al., 2020) image data in diverse areas of medical research. This review summarizes radiomics methodology and critically synthesizes spine applications across osteoporosis, vertebral fracture, low back pain, intervertebral disc disease, and metastatic spine disease. To improve readability for both clinical and technical audiences, the review is organized in two complementary parts. Section 2 provides methodological background for readers seeking details on the radiomics workflow and model development. Section 3 focuses on clinical applications for readers primarily interested in spine disease areas, imaging modalities, and translational relevance.

## 2 Radiomics: methods and model building

**FIGURE 1:**
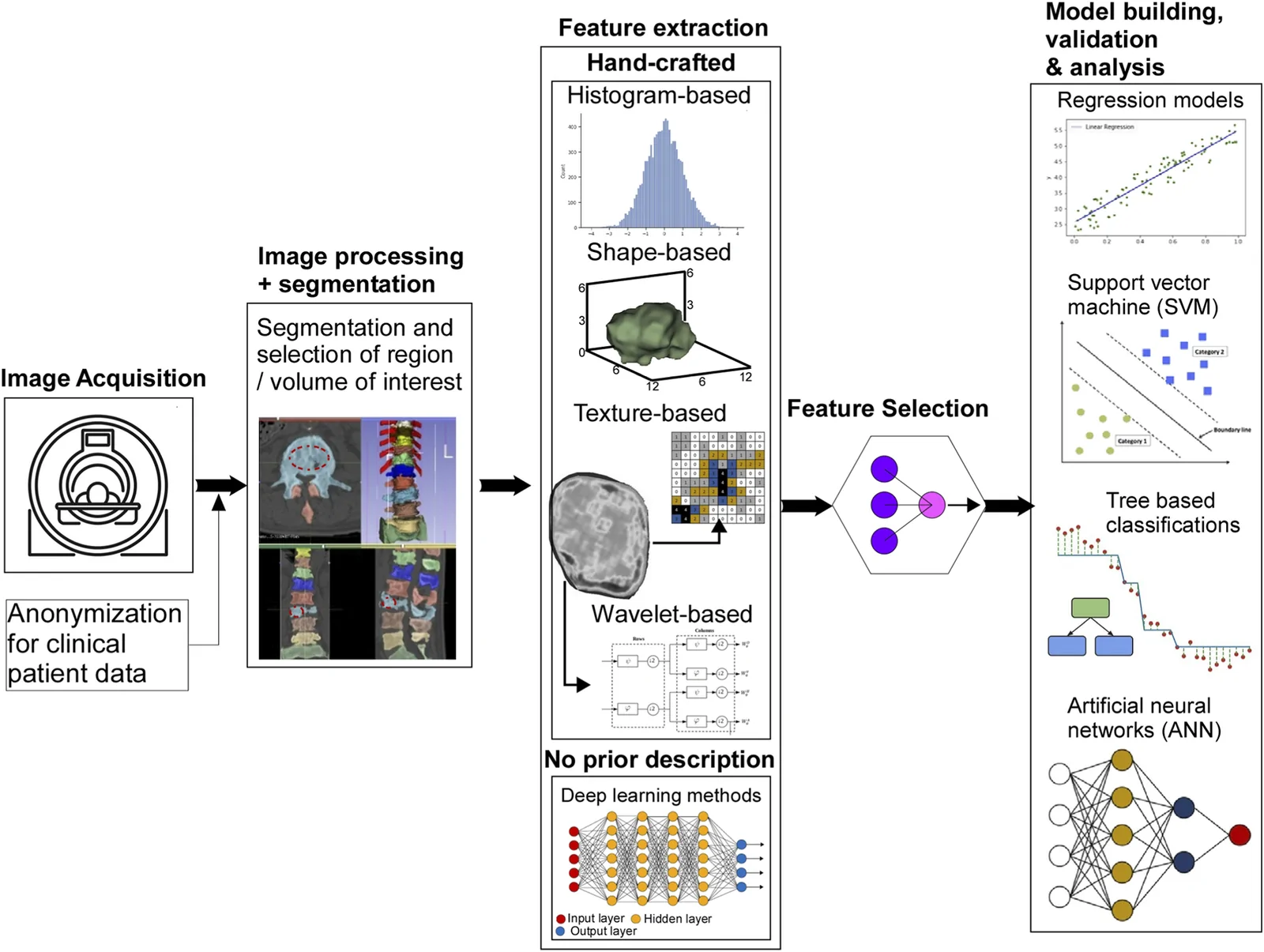
Illustration of the radiomics workflow. Image data acquired using clinical or research scanners is anonymized (clinical data), the data is prepared, and segmentation is applied to extract the tissue/anatomy of interest. Extraction of radiomics features (hand-crafted or deep learning based) is performed, followed by model selection using machine learning or deep learning approaches, followed by model development, validation and analysis.

The general radiomics workflow commonly consists of four stages: 1) image preprocessing, 2) image segmentation, 3) feature extraction and 4) feature analysis (Figure 1) (van Timmeren et al., 2020).

### 2.1 Image preprocessing

The stability and repeatability of radiomics analysis are significantly affected by image acquisition parameters (pixels (2D) or voxels (3D) size, gray level values) and reconstruction algorithms (Aerts et al., 2014; Ramlee et al., 2024; Wichtmann et al., 2023). Preprocessing stages: segmentation, intensity normalization, coregulation, noise filtering and pixel binning are analytical techniques crucial for improving image quality and ensuring statistical repeatability and comparability (Wichtmann et al., 2023) with the exact series of operations being imaging modality dependent. The most widely used open-source tool for automated feature extraction is PyRadiomics (van Griethuysen et al., 2017), providing data pre-processing and batch extraction options and a 3D Slicer plug-in (https://github.com/AIM-Harvard/SlicerRadiomics), for graphical user interface-based selection and computation (Fedorov et al., 2012).

### 2.2 Harmonization

Multi-center data frequently exhibit batch effects arising from variability in imaging hardware (scanner manufacturer, magnetic field strength, scanner drift, hardware imperfections) and associated acquisition parameters (Badhwar et al., 2020; Poldrack and Gorgolewski, 2014). These differences can give rise to undesirable batch effects. Aggregating small imaging datasets can reduce statistical power, diminish replicability, and introduce bias affecting model performance (Bell et al., 2022; Carre et al., 2022). Image resampling or filtering (Mackin et al., 2017) may reduce inter-scan variability but at the cost of spatial resolution (Mackin et al., 2017). Moreover, even when acquisition properties are tightly controlled, such as in single-scanner studies, hardware imperfections, operator and site characteristics, and software or hardware upgrades in long-running studies can introduce batch effects (Jovicich et al., 2016; Shinohara et al., 2017). Techniques such as ComBat (Horng et al., 2022) can estimate and account for additive batch effects (affecting measurements) and multiplicative batch effects (affecting the measurement error term). In cancer patients, these methods were shown to reduce diverging feature distributions in MR- and CT PET radiomics studies (Mahon et al., 2020; Orlhac et al., 2022).

### 2.3 Segmentation

Segmentation defines the 2D region of interest (ROI) or 3D volume of interest (VOI) from which features are extracted. Historically, ROIs/VOIs were contoured manually or semi-manually by a trained observer or expert clinician (Heckel et al., 2014) from imaging data (CT, MRI, PET, Ultrasound), ideally by more than one person to offset bias. Semi-automated approaches such as GrowCut (Wallner et al., 2018), region growing (Tamez-Pena et al., 1999), and fuzzy logic (Sasaki et al., 1999). Increasingly, machine learning (ML) (Misir, 2025) and deep learning (DL) networks (Bonaldi et al., 2023; Chen Y. et al., 2024; Madzia-Madzou et al., 2025; Saeed et al., 2023; Tao et al., 2022; Wasserthal et al., 2023; Diaz-Pinto et al., 2022; Haouchine et al., 2024), such as MONAI (Diaz-Pinto et al., 2022; Diaz-Pinto et al., 2024) and nnUNet (Isensee et al., 2021), offer automated high-throughput segmentation with accuracy equal to or exceeding expert-based segmentation (Reznikov et al., 2020; Rich et al., 2023) with superior reproducibility through controlled observer variability (Haarburger et al., 2020).

The spine is a complex multi-articular system comprising vertebral bodies, intervertebral discs (IVDs), spinal ligaments, and spinal and paraspinal musculature. Segmentation of the vertebrae and IVDs faces unique challenges:

#### 2.3.1 Anatomical complexity and variability

Vertebral and IVD anatomy is intricate, particularly the posterior elements, which vary markedly in size, shape and geometry both within and between individuals. Transitional level variations: T13, L6 and lumbosacral transitional vertebrae, present in up to 16% of the population (Chiu et al., 2024), result in inaccurate automated level labeling for algorithms trained on standard 7 cervical, 12 thoracic, 5 lumbar and 5 sacral distributions.

#### 2.3.2 Vertebral pathology

Bone metastasis, degenerative vertebral and IVD changes, and other pathologies substantially challenge automated segmentation.

##### 2.3.2.1 Spinal bone metastases

Spinal bone metastases, occurring in 30%–70% of cancer patients, cause significant morbidity through pain, neurological compromise and pathological fractures, producing marked variation in vertebral bone structure (Bailey et al., 2020; Bailey et al., 2022) and the destruction of vertebral anatomy (Coleman et al., 2020). Osteolytic lesions exhibit decreased attenuation relative to normal trabecular bone due to loss of bone density and/or architectural destruction. Distinguishing lytic changes from normal age-related heterogeneity and conditions such as osteoporosis is difficult unless large, discrete foci are present. However, not all spinal lesions are neoplastic (McCullagh et al., 2023): demyelinating lesions, vascular lesions including vertebral body hemangiomas (affecting 1.9%–27% of the population (Jiang et al., 2014), and inflammatory or infectious lesions such as tuberculosis can mimic malignancy (McCullagh et al., 2023). Osteoblastic lesions are frequently heterogeneous and may coexist with osteolytic lesions (mixed lesions), rendering a unified segmentation approach highly challenging.

CT and MRI provide complementary information: CT excels at cortical bone destruction and cancellous bone calcification assessment, while MRI offers superior sensitivity for bone marrow infiltration, tumor extent and epidural extension. Combining these modalities is complicated by differences in acquisition protocols, spatial resolution and contrast mechanisms that hinder cross-modal alignment and standardization (Section 2.2) (Faiella et al., 2022). Notably, the benchmark VerSe segmentation challenge (MICCAI 2019/2020) explicitly excluded traumatic fractures and bony metastases, underscoring the challenge of pathological spine segmentation (Diaz-Pinto et al., 2022; Haouchine et al., 2024). Despite growing radiomics and DL capabilities for automated segmentation and malignancy grading, clinical deployment remains limited by interoperability issues and the absence of standardized preprocessing pipelines (Chang et al., 2022; Huo et al., 2025).

##### 2.3.2.2 Intervertebral disc (IVD)

In healthy IVDs, the hydrated nucleus pulposus (NP) exhibits a high T2 signal, enabling contrast-based delineation from the surrounding annulus fibrosus (AF) and vertebral endplates (Pfirrmann et al., 2001). With aging and degeneration, this NP–AF signal contrast diminishes and is largely eliminated in advanced stages (Pfirrmann et al., 2001), challenging all segmentation pipelines (Zheng et al., 2022). Advanced degeneration also causes structural remodeling: disc height loss, endplate irregularities, osteophyte formation, and IVD failure by herniation or NP extrusion. These morphological alterations further degrade sub-compartment segmentation of the NP and AF required for clinical and biomechanical modelling rather than whole-disc delineation (Matos et al., 2023). Deviating substantially from normal training distributions, such changes produce boundary delineation errors and are consistently identified as the most challenging failure mode for DL segmentation models (Hong et al., 2026).

#### 2.3.3 Clinical imaging

Partial volume effects (PVEs) arise when image resolution cannot resolve intended tissue details, causing “fusion” of anatomical structures at the pixel (2D) or voxel (3D) level, blurring tissue boundaries and generating image elements that represent a mixed average of multiple tissue properties. In clinical CT, typical spatial resolution (1–2 mm thick slices, 0.65–0.9 mm in-plane) is insufficient to separate the thin cortical shell (≤500 μm) from cancellous bone, may prevent facet joint space delineation, and in advanced degeneration can cause adjacent vertebral body “fusion,” producing mis-segmentation and level mislabeling. The recent introduction of clinical photon-counting CT (PCCT), enabling improved image resolution (0.15–0.25 mm thick slices, 0.1–0.3 mm in-plane) (Willemink et al., 2018), superior noise-reducing capacities (Yang Y. et al., 2025) and energy-weighting reconstruction algorithms, was reported to significantly improve quantifying bone mechanical anisotropy (Quintiens et al., 2026; Quintiens et al., 2025). Uniquely, multi-energy (spectral) data acquisition allows detection of bone marrow edema associated with fractures (Ventura et al., 2024), may aid in the detection of degenerative disease and metastatic changes in bone quality and structure, offering new and improved clinical fracture risk predictions and patient management. In MRI, anisotropic spatial resolution, bias field artifacts, and the absence of standardized measurement units compound PVE (Hille et al., 2018; Stern et al., 2011), impairing NP and AF segmentation due to blurred tissue boundaries (Silvoster et al., 2020). Image noise, hardware acquisition artifacts, low signal-to-noise ratio, and insufficient spatial resolution may force segmentation algorithms to rely heavily on prior anatomical models to resolve structural boundaries from blurred voxel values (Korez et al., 2015). Post-surgical metal hardware may create image artifacts or prevent the ability to acquire image data due to hardware incompatibility, a challenge in MR and CT imaging (Do et al., 2018)^.^

#### 2.3.4 Limited and imbalanced training data

The scarcity of large, well-annotated public datasets is a fundamental limitation in developing robust automated segmentation algorithms. Expert annotation of spinal metastases is laborious and requires subspecialty radiological expertise, yielding datasets that are typically small and institution-specific. Manual segmentation, which serves as ground truth for supervised learning, is subject to considerable inter-observer variability. Class imbalance presents a further challenge: metastatic voxels constitute a small fraction of the total image volume in whole-spine CT or MRI, creating foreground/background imbalance that biases models toward background prediction (Cui et al., 2025; Naseri et al., 2023).

The clinical validity of fully automated segmentation remains an open question, and expert ROI validation remains a necessary step in the radiomics segmentation pipeline. A more thorough discussion of these developments is found in the following reviews (Do et al., 2018; Elliott et al., 2026; Patel et al., 2025; Sanker et al., 2025; Wang et al., 2025; Yoo, 2025; Stein et al., 2023).

### 2.4 Feature extraction

**FIGURE 2:**
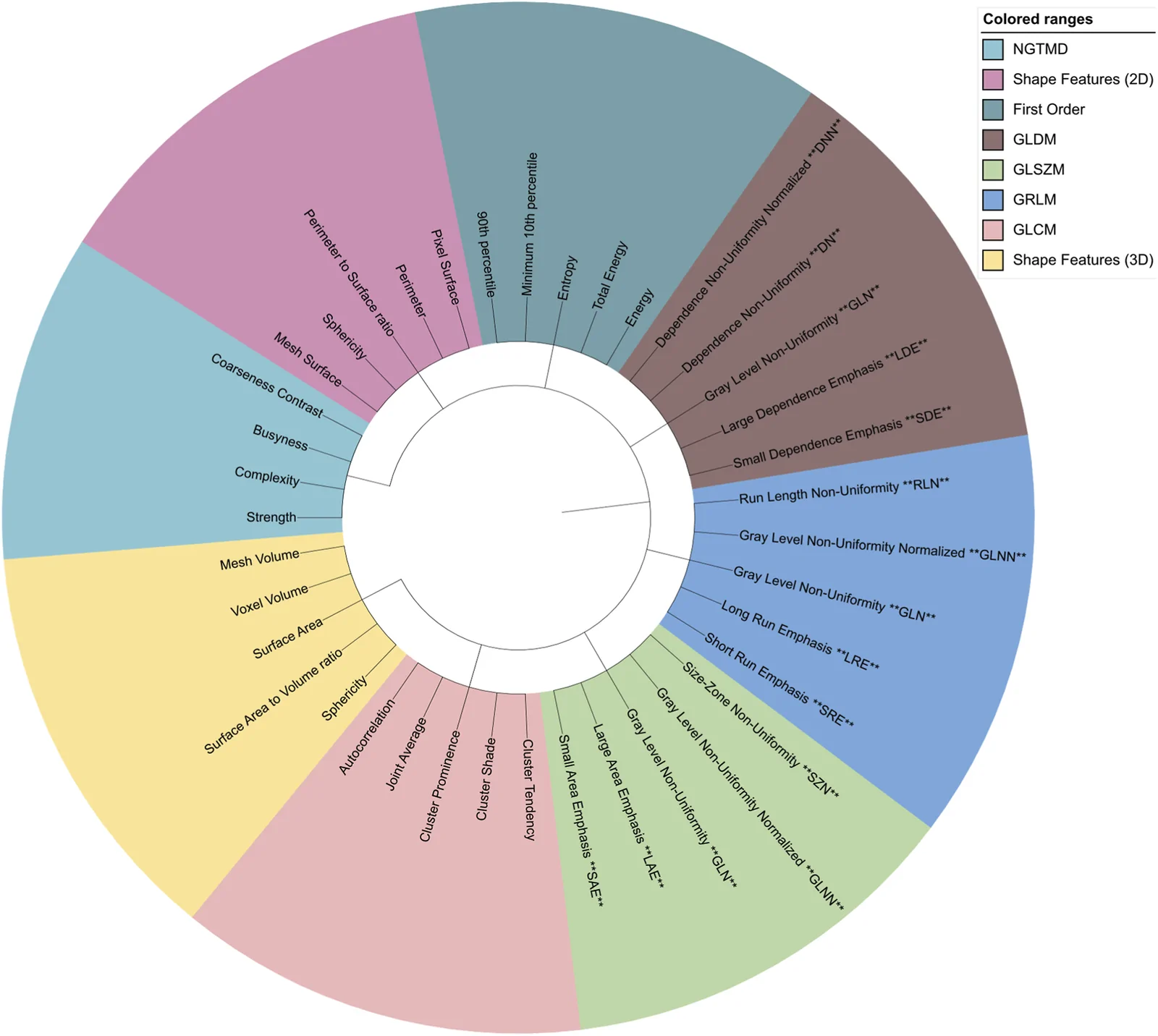
A phylogenetic tree illustrating radiomic features computable by the PyRadiomics library, grouped by their class. Identified by a color with a key for the class, each class is aggregated based on the Image Biomarker Standardization Initiative (IBSI). For clarity, we include 5 features per category of radiomics features. Full list is provided by PyRadiomics (van Griethuysen et al., 2017).

This process employs software-based, pre-determined, numerical (handcrafted) functions (Liu et al., 2021) to compute quantitative descriptors for radiomics analysis. The Image Biomarker Standardization Initiative (IBSI) (Zwanenburg et al., 2020) classifies these into five categories of increasing complexity, Figure 2.

**FIGURE 3:**
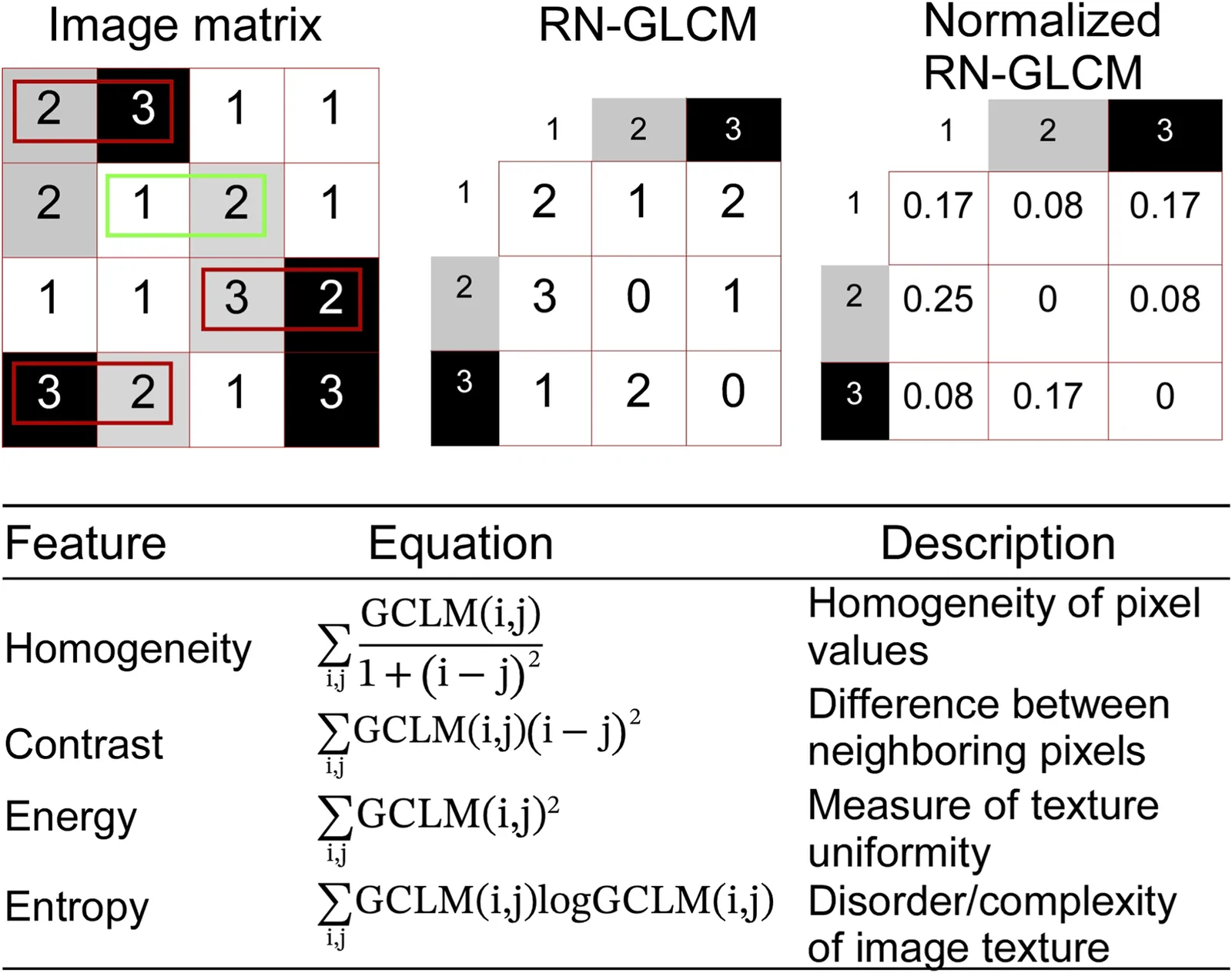
Right neighbor GLCM (RN-GLCM), computed by counting the number of qualifying pairs of pixel values, followed by normalizing by the overall sum, is used to derive textural parameters from standardized equations. Pixel pairs are highlighted in green [1,2] and red [2,3].

1. Intensity-based features are derived from the statistical distribution of pixel or voxel intensities within an ROI/VOI. First-order statistics (mean, variance, skewness, kurtosis) characterize overall intensity without spatial context. In PET, such features relate to F-fluorodeoxyglucose (FDG) tracer uptake, enabling quantification of lesion avidity to assess malignant potential (Nwogu and Corso, 2008).
2. Shape-based features describe 2- and 3-dimensional geometric and morphological forms, including size, min/max diameters, volume, surface area, and compactness or sphericity. For example, whether a tumor is round, polygonal, or spiculated, is a strong indicator of benignity (Snoeckx et al., 2018).
3. Texture-based features are second-order statistics derived from matrices such as the Gray-Level Co-occurrence Matrix (GLCM) and Gray-Level Run-Length Matrix (GLRLM), quantifying spatial intensity relationships. Parameters including homogeneity, contrast, energy and entropy are computed from the normalized GLCM via standardized equations (Figure 3). In cancer, textural features likely reflect intra-tumoral heterogeneity and have been associated with clonal subpopulations driving tumor growth and treatment resistance (Davis et al., 2017).
4. Model-based features involve fitting mathematical models to intensity data to describe complex spatial patterns. Fractal analysis, for example, quantifies the complexity and self-similarity of target structures, providing insight into irregular tissue organization.
5. Transform-based features, such as those derived by Fourier or wavelet transforms, highlight the (spatial-) frequency components of the image, revealing repeating spatial relationships or details at multiple scales and orientations not apparent in the spatial domain. The Fourier transform represents the spatial harmonics of the original image. The wavelet transform retains both scale and location information at the expense of additional complexity. The choice of transform depends on the image structure and the feature class best suited to the intended downstream processing. For example, detecting abrupt intensity transitions between osteolytic and osteoblastic lesions in mixed spinal metastatic disease, common in prostate and breast cancer patients (Bailey et al., 2022). Together, these five categories enable comprehensive medical image analysis, supporting improved lesion characterization and personalized imaging approaches.

### 2.5 Model development

Following feature extraction and normalization, data are divided into training and internal validation sets balanced for patient factors such as age, performance status and disease characteristics. Model development is divided into statistical or DL-based approaches.

#### 2.5.1 Model development

Current approaches for model development can be:

##### 2.5.1.1 Statistical methods

In parametric modelling, a functional form of the radiomics prediction vector (RPV) is a sum of features *x*_1_ … *x*_*n*_ with weights *β*_1_ … *β*_*n*_ (Equation 1).

$$RPV={\;\beta }_{1}{\;x}_{1}+{\;\beta }_{2}{\;x}_{2}+....\;{+\beta }_{n}{\;x}_{n}$$ (1)

Non-significant features are removed based on a false detection rate (FDR) threshold (typically 5%), followed by correlation analysis to exclude collinear features.

Feature selection methods based on training on known outcomes (“labelled data”) are considered examples of supervised learning (Table 1). Such methods include: Cox or logistic regression models are used for the prediction of survival or binary outcomes. Naïve Bayes, calculates probabilistic priors using Bayesian framework requiring smaller training samples to attain good performance. However, it is limited by its assumption of independence of features (Rish, 2001). Non-parametric methods (Avanzo et al., 2020; Wu et al., 2016) provide a more general approach. K-nearest neighbors (KNN) classify samples based on the distance of a data point to its nearest neighbors in feature space, but is prone to overfitting in high-dimensional radiomics settings (Wang and Hu, 2005). Random Forests (RFs) build ensembles of decision trees that vote on classification or regression outcomes (Breiman, 2001), assigning importance scores via node impurity reduction and handling non-linear relationships and feature interactions in complex imaging data. This technique is advantageous as it handles both non-linear relationships and interactions among features commonly found in complex imaging data. Support vector machine (SVM) computes the separating hyperplane between data points, using kernel transformations allowing non-linear classification (Wang and Hu, 2005).

**TABLE 1:** Machine learning algorithms in medical imaging.

Model	Algorithm	References
Linear learning	Linear regression	Nelder and Wedderburn (1972)
	Logistic regression	Walker and Duncan (1967)
	Cox regression	Cox (1972)
	Principal component analysis (PCA)	Jolliffe (2006)
	Linear discriminant analysis (LDA)	McLachlan (2005)
	Ridge regression	Hoerl and Kennard (1970)
	Least absolute shrinkage and selection operation (LASSO)	Tibshirani (1996)
	Elastic net regression	Zou and Hastie (2005)
Nonlinear learning	Naïve bayes (NB)	Russell and Norvig (2021)
	General additive models (GAM)	Hastie (2009)
	Decision tree	Quinlan (1987)
	Random forest	Ho (1998)
	Gradient boosting machine (G-Boost)	Hastie (2009)
	Advanced gradient boosting (XG-Boost)	Chen and Guestrin (2016)
	Support vector machine (SVM)	Cortes and Vapnik (1995)
	Artificial neural network (ANN)	Kleene (1951)
	K-nearest neighbors (K-NN)	Fix (1951)
	Deep learning (DL)	Alzubaidi et al. (2021)

Unsupervised methods analyze unlabeled data solely from input structure include: K-means clustering, which performs data separation based on the geometric distance of data points to cluster centroids (Sinaga and Yang, 2020), and Principal Component Analysis (PCA) that transforms data into uncorrelated principal components capturing maximum variance (Abdi and Williams, 2010); PCA is less prone to overfitting than supervised models due to the inclusion of feature interaction effects (Zhang et al., 2017).

#### 2.5.2 Model optimization

An optimal model balances bias and variance (Carrasquinha et al., 2019). Ridge regression penalizes squared coefficient estimates (Hoerl and Kennard, 1970), shrinking less important variables without eliminating them. Least absolute shrinkage and selection operation (LASSO) forces coefficients of less important features to zero via an absolute value penalty (Tibshirani, 1996), reducing multicollinearity and enhancing interpretability, particularly valuable when hundreds to thousands of features are extracted. Elastic net regression (Zou and Hastie, 2005), particularly useful when dealing with highly correlated features, balances between L1 and L2 regularization, is especially suited to highly correlated features, retaining informative correlated groups rather than eliminating them, highly beneficial in spine imaging where anatomical and physiological interdependencies may manifest in correlated radiomic feature selection (Ge and Zhang, 2023).

##### 2.5.2.1 Deep learning (DL) radiomics

**FIGURE 4:**
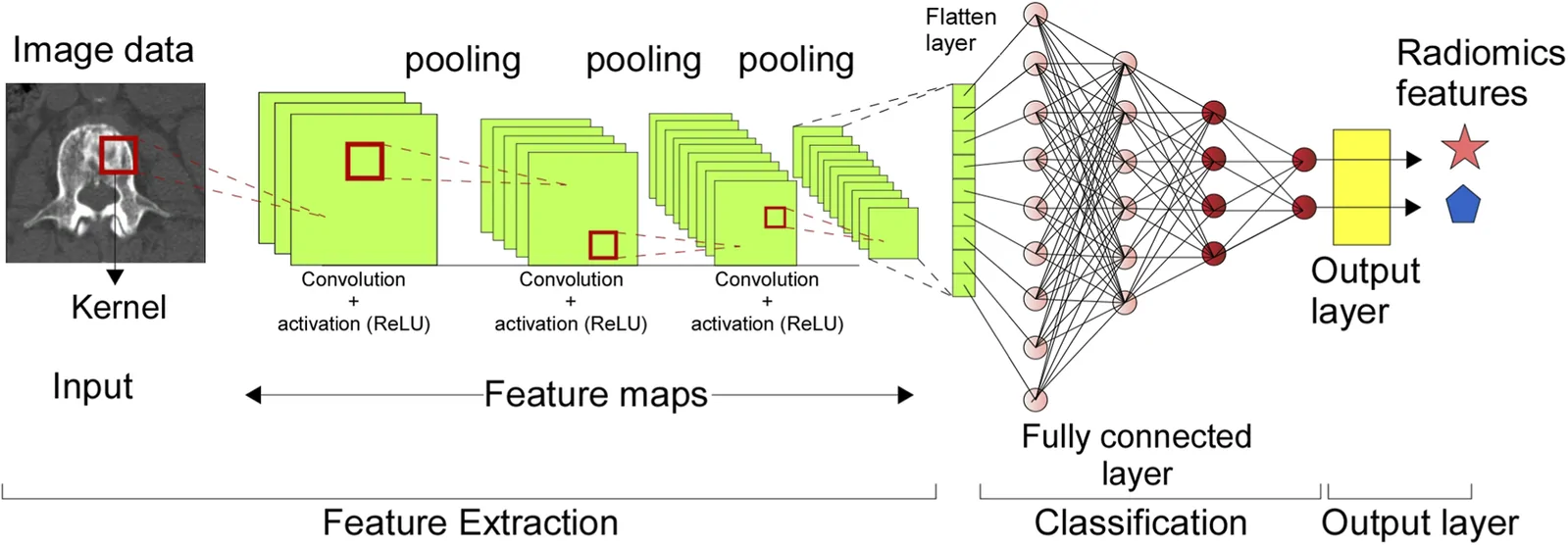
An example of the architecture of a CNN for image classification, which consists of convolutional layers and fully connected layers of classical artificial neurons.

Traditional radiomics pipelines require accurate segmentation and handcrafted feature extractors as classifier inputs (Hosny et al., 2019), typically generating >1,000 highly correlated features per region, increasing redundancy and overfitting risk, particularly in small or moderate spine study samples. DL-based radiomics instead learns features automatically and directly from imaging data, reducing reliance on handcrafted descriptors and, in some cases, explicit segmentation (Lou et al., 2019; Liu et al., 2024). Dimensionality reduction is performed implicitly through convolution, pooling and feature aggregation, producing compact latent representations without requiring the explicit feature selection or *post hoc* reduction steps of traditional pipelines. CNNs process fixed-size cropped image patches or volumes through multi-layer networks of millions of parameters estimated during training (Figure 4), learning hierarchical representations that capture local texture, mid-level structural organization and higher-level contextual information.

- Feature detection stage: Convolution, nonlinear activation (e.g., ReLU) and pooling operations encode the input image into progressively lower-dimensional feature maps (Figure 4), implicitly modelling complex spatial relationships and multi-scale patterns difficult to capture with predefined radiomic descriptors.
- Classification stage: The feature maps are flattened into a unidimensional array and passed to a fully connected layer producing class probability outputs (Figure 4). Feature extraction and classification are jointly optimized end-to-end, directly aligning learned features with the clinical prediction objective.

Networks may be built from scratch or initialized from pre-trained models updated via transfer learning; the latter is more common given typical dataset size constraints. Backpropagation and optimization algorithms, such as gradient descent, iteratively adjust parameters to minimize prediction error during training. Despite these stated advantages, key limitations include large data requirements (hundreds to thousands of images), with both training and validation being greatly dependent on the quality of clinical data used for training labels (Qin et al., 2018), affecting DL model’s generalizability (Demircioglu, 2025). Significantly, the limited interpretability of the resulting model (“black box”) results in great difficulties in the physical interpretation of the CNN model. Visualization techniques such as activation or saliency mapping partially address this by generating heatmaps from the final convolutional layer to highlight regions most influential in model predictions, which can then be radiologically interpreted (Chattopadhay et al., 2018).

### 2.6 Model validation

Validation assesses overfitting and determines whether a model generalizes beyond its development dataset. Strategies are broadly categorized as internal or external (Rizzo et al., 2018; Steyerberg and Harrell, 2016). External validation, testing a finalized model on an independent dataset from a different institution, time period or population (Steyerberg and Harrell, 2016), provides the strongest evidence of generalizability and clinical robustness, as it evaluates model performance under conditions that more closely resemble real-world deployment (Rizzo et al., 2018; Jha et al., 2023). Internal validation applies resampling or partitioning within the original dataset (Steyerberg and Harrell, 2016; Eertink et al., 2022) and reduces optimism bias during development, but does not fully address dataset-specific overfitting (Eertink et al., 2022; Collins et al., 2015). Common internal strategies include Holdout validation (training/validation/testing split) (Bradshaw et al., 2023; Kohavi, 1995), K-fold cross-validation (Hastie et al., 2009; James et al., 2013), and Leave-one-out cross-validation (LOOCV) (Stone, 1974).

Importantly, internal cross-validation metrics are not equivalent to external validation performance (Steyerberg and Harrell, 2016; Eertink et al., 2022): cross-validation measures internal predictability, whereas external validation assesses transportability across populations and imaging environments (Rizzo et al., 2018; Steyerberg and Harrell, 2016). Clear reporting of validation methodology is thus essential for interpreting model credibility and clinical readiness. Therefore, models supported only by internal validation or cross-validation should be considered hypothesis-generating rather than clinically ready, and reported AUC values from these designs should not be interpreted as evidence of transportability across institutions, scanners, or patient populations. In spine radiomics, this distinction is particularly important because models are often developed from small or single-institution datasets with limited variation in scanner type, acquisition protocol, reconstruction method, patient population, and disease severity. Under these conditions, internal validation may confirm that a model performs well within the development dataset, but it does not establish that the model will perform similarly when applied to images from another hospital, scanner vendor, MRI sequence, CT reconstruction kernel, or clinical workflow. External validation should therefore be considered a minimum requirement before a radiomics model is described as clinically generalizable. Future studies should also report calibration, confidence intervals, decision-curve analysis, and performance across clinically relevant subgroups, rather than relying only on AUC values.

### 2.7 Reproducibility

PyRadiomics (van Griethuysen et al., 2017) extracts up to 1,500 features per image, many of which capture overlapping information, for example, variance and standard deviation among intensity features, or sphericity and spherical disproportion among shape features. Including such redundant features introduces unnecessary bias, increases overfitting risk, and reduces generalizability to new datasets. Image acquisition, reconstruction and segmentation (Section 2.3) each influence reproducibility (Zhang et al., 2022). The calculation of the intra-class correlation coefficient (ICC), computed for test-retest, repeated scan acquisition or multi-observer segmentation techniques (Xue et al., 2021), identifies unreliable features; a threshold of 0.75–0.90 is typically applied to exclude them.

### 2.8 Open-source data for spine radiomics

Publicly accessible datasets support assessment of acquisition parameter effects on radiomics robustness (Ronneberger et al., 2015), enable identification and minimization of non-robust radiomic characteristics, and facilitate development of pathology-targeted models. Further work is needed to create and curate such databases across MRI, CT and PET-CT spine imaging modalities.

## 3 Application of radiomics for evaluating spinal osteoporosis, pathology and fracture

The primary objective of this narrative review was to evaluate the application of radiomics in the spine. First, we present the concept and key feature classes of radiomics, data preparation, segmentation, and application of statistical methods and deep learning for developing radiomics-based models. We then discuss the application of radiomics for assessing osteoporosis, low back pain (LBP) and intervertebral disc disease, bone metastasis and benign and malignant vertebral fracture risk. The review culminates with the presentation of the current limitations and future research directions, opportunities for integration of radiomics with multi-omics analysis, highlighting radiomics’ potential as a surrogate biomarker in the fields of spinal biomechanics. We included these domains as each relies on quantitative characterization of vertebral bone, marrow, intervertebral disc, or lesion heterogeneity from routine spine imaging. We organized our synthesis by clinical target, imaging modality, and model type to clarify the relationship between osteoporosis, vertebral fracture, low back pain, intervertebral disc disease, and metastatic spine disease.

A structured comparison across these disease areas also highlights several cross-cutting patterns. CT-based radiomics has been most frequently applied to osteoporosis, vertebral fracture assessment, and lesion characterization because CT provides relatively standardized attenuation values and high spatial resolution for cortical and trabecular bone. MRI-based radiomics is more commonly used for intervertebral disc degeneration, low back pain, marrow infiltration, and metastatic disease because MRI provides superior soft-tissue and bone marrow contrast. However, MRI radiomics is also more sensitive to sequence selection, scanner hardware, field strength, intensity normalization, and preprocessing choices. Across disease areas, traditional handcrafted radiomics models remain more interpretable but depend heavily on segmentation and feature selection, whereas deep learning and hybrid models may improve classification performance at the cost of interpretability, larger data requirements, and greater validation burden.

### 3.1 Methodology

#### 3.1.1 Literature search strategy

A narrative literature review was conducted to summarize current concepts and applications of radiomics in spine imaging, specifically regarding the domains of: 1) Assessment of Osteoporosis in the Spine. 2) Prediction of Fragility Vertebral Fractures, 3) Chronic Low Back Pain, 4) Assessment of Intervertebral Disc Degeneration, 5) Intervertebral Disc Herniation Detection and Classification, 6) Cancer Metastatic Spine Disease, 7) Differentiation of Benign vs. Malignant Lesions and 8) Diagnostic Classification of Benign vs. Malignant Vertebral Fractures. Relevant publications were identified through searches of PubMed and Embase and through review of reference lists from key articles. Our search strategy involved using a grouping of keywords and Medical Subject Headings (MeSH) to ensure wide-ranging coverage. Specifically, we queried the databases with the search term: [“radiomics” AND [“osteoporosis” OR “fracture” OR “low back pain” OR [“intervertebral disc” AND [“degeneration” OR “herniation”]] OR “metastatic spine disease” OR “lesion”]] with a date range from 2016 (the year the seminal work by Gillies et al. (Gillies et al., 2016) was published) to the date of the query (30 June 2025).

#### 3.1.2 Study screening and selection criteria

Each selected study was reviewed based on radiomic feature extraction and analysis techniques, imaging modalities used, study design, clinical outcomes evaluated, and the overall impact of radiomics on spinal fracture risk assessment, osteoporosis diagnosis, evaluation low back pain and intervertebral disc degeneration and diagnosis of metastatic spine disease and differentiation of benign vs. malignant fractures. Special attention was paid to methodological aspects such as image acquisition protocols, comprehensive feature selection methods, and statistical analysis.

#### 3.1.3 Data extraction and reporting

The data extracted from the selected sources were organized into the previously detailed (Section 3.1.1) thematic categories relevant to the review’s focus. Information gathered from each research article included:

- Details of the research article: authors, publication date, journal name.
- Prediction target(s).
- Details regarding model creation: training cohort size, mode of validation, validation cohort size, and model performance.

For each theme, narrative synthesis provides a cohesive overview of reported methods, tools, outcomes, and domain-specific radiomics challenges.

### 3.2 Assessment of osteoporosis in the spine

**FIGURE 5:**
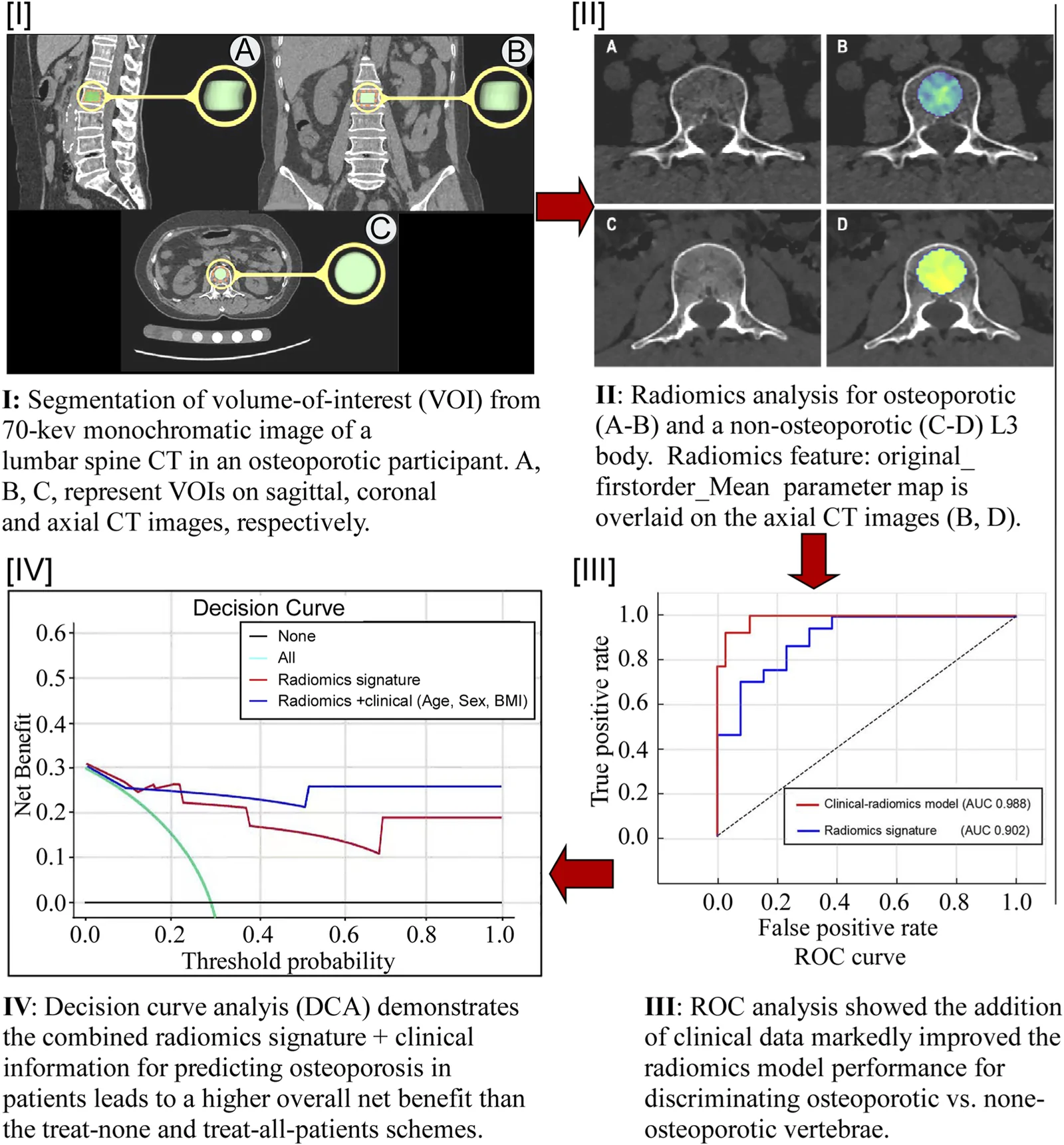
Application of radiomics modeling demonstrates improved prediction of vertebral osteoporosis in patients from dual-energy CT monoenergetic imaging. Incorporation of clinical demographic parameters further improves the prediction. Image adapted from Wang J. et al. (2023), BMC Musculoskeletal Disorders (2023) 24:100.

The World Health Organization (WHO) defines osteoporosis as a systemic skeletal disease characterized by low bone mass and deterioration of bone tissue microarchitecture, consequently increasing bone fragility and susceptibility to fractures (Genant et al., 1999) (Table 2). Type I osteoporosis, the most frequent type, affects mainly women between 51 and 75 years of age, characterized by rapid bone loss (Jiménez et al., 2009). Senile osteoporosis (Type II), characterized by trabecular and cortical bone loss, affects subjects aged >75 years (Sozen et al., 2017). Secondary osteoporosis, following disease or medication use, accounts for <5% of osteoporosis cases (Khosla et al., 1994). He et al. (2021), pioneering the application of radiomics for predicting spinal osteoporosis defined based on WHO criteria in 109 patients, demonstrated handcrafted textural radiomics features, indicative of MR signal changes and bone marrow and structure heterogeneity, distinguished normal bone, osteopenia, and osteoporosis (AUC 0.682–0.810). Xue et al. (2022) extracted 1,197 handcrafted radiomic features from lumbar spine CT images of 133 patients, applied a machine learning approach for model selection (Support vector machine (SVM), random forest (RF), and K-nearest neighbor (KNN)). The model found radiomics features reflecting higher complexity and heterogeneity of the bone structure to strongly distinguish normal BMD vertebrae vs. osteoporotic vertebrae (AUC: 0.994), osteopenic vs. osteoporotic vertebrae (AUC: 0.866), and normal BMD vs. osteopenic vertebrae (AUC: 0.940). Applying machine learning (LASSO) for model selection, shape and textural handcrafted radiomics features extracted from T1-weighted (T1WI) and T2-weighted (T2WI) MR data were demonstrated to strongly differentiate osteoporosis vs. non-osteoporosis subjects selected based on Dual-Energy X-ray Absorptiometry (DEXA) imaging (Kang and Wang, 2024; Zhen et al., 2024). Using a DL model to address the segmentation challenges, Liu et al. (2024) found radiomics features from the trabecular and vertebral body to strongly discriminate between vertebral osteoporosis and non-osteoporosis (AUC: 0.97), Figure 5. Vertebral bone mineral density (BMD), typically assessed via DEXA, remains the gold-standard clinical measure for diagnosing osteoporosis (Blake and Fogelman, 2007). However, this scalar metric cannot capture the microarchitectural changes underlying osteoporosis-related loss of vertebral strength (Keaveny et al., 2001). CT-based radiomics models—incorporating first-order, shape, textural, and wavelet features—have demonstrated higher precision than DEXA in predicting BMD, achieving AUCs of 0.92 vs. 0.86 in a 99-patient study (Jiang et al., 2022) and an *R* ^2^ of 0.83 (Pearson correlation: 0.93) using LASSO-selected features in a 245-patient study (Dai et al., 2023). In a 429-patient cohort imaged with lumbar MRI, radiographs, and DEXA within 6 months, radiomics showed good discriminative performance for detecting low BMD (T-scores −1 to −2.5), though the authors noted limited capability for direct BMD value estimation (Galbusera et al., 2024). These studies support the feasibility of radiomics not only for osteoporosis classification but also for predicting bone quality metrics, advancing the potential for comprehensive osteoporosis assessments (Figure 5).

**TABLE 2:** List of all osteoporosis models.

References	Title	Prediction target(s)	Model features	Training cohort	Mode of validation^#^	Validation/test cohort	Performance (AUC-ROC)
Chen B. et al. (2024)	Application of radiomics model based on lumbar computed tomography in the diagnosis of elderly osteoporosis	Elderly Osteoporosis	CT radiomics	127	Internal	55	0.83
Cheng et al. (2023)	A diagnostic approach integrated multimodal radiomics with machine learning models based on lumbar spine CT and X-ray for osteoporosis	Osteoporosis	CT radiomics + Demographics, + BMI + ALP, and Ca^2+^ levels)	431	Internal	185	0.91
Cheng et al. (2024)	Enhancing the Opportunistic Bone Status Assessment Using Radiomics Based on Dual-Energy Spectral CT Material Decomposition Images	Normal vs. Osteoporosis vs. Osteopenia	Spectral DECT radiomics	214	Internal	93	0.90
Fang et al. (2024)	A comprehensive approach for osteoporosis detection through chest CT analysis and bone turnover markers: harnessing radiomics and deep learning techniques	Osteoporosis vs. osteopenia	CT radiomics + demographics	392	Internal	96	0.91
Galbusera et al. (2024)	Estimating lumbar bone mineral density from conventional MRI and radiographs with deep learning in spine patients	Osteoporosis and Osteopenia	MRI radiomics + demographics	373	Internal	86	0.87
He et al. (2021)	Radiomics based on lumbar spine magnetic resonance imaging to detect osteoporosis	Osteoporosis	MRI radiomics	109	3-fold cross-validation	NA	0.80
Jiang et al. (2022)	Radiomics analysis based on lumbar spine CT to detect osteoporosis	Lumbar spine osteoporosis	CT radiomics	270	Internal	116	0.92
Lin X. et al. (2024)	Utilizing radiomics techniques to isolate a single vertebral body from chest CT for opportunistic osteoporosis screening	Osteoporosis	CT radiomics + demographics	545	External	242	0.81
Liu et al. (2024)	Hybrid transformer convolutional neural network-based radiomics models for osteoporosis screening in routine CT	Osteoporosis	CT radiomics	204	Internal	79	0.97
Mohammadi-Sadr et al. (2025)	A novel approach based on integrating radiomics, bone morphometry and hounsfield unit-derived from routine chest CT for bone mineral density assessment	Elderly osteoporosis	CT radiomics	127	5-fold cross-validation	NA	0.93
Pan J. et al. (2024)	Feasibility study of opportunistic osteoporosis screening on chest CT using a multi-feature fusion DCNN model	Osteoporosis	CT radiomics + demographics + BMI + laboratory biomarkers^1^	1048	External	537	0.99
Pan Y. et al. (2024)	Radiomics models based on thoracic and upper lumbar spine in chest LDCT to predict low bone mineral density	Low BMD	LDCT radiomics	622	Internal	283	0.89
Wang J. et al. (2023)	Prediction of osteoporosis using radiomics analysis derived from single-source dual-energy CT	Osteoporosis of the Spine	CT radiomics + demographics + BMI	114	Internal	50	0.99
Wang J. et al. (2024)	Predicting osteoporosis and osteopenia by fusing deep transfer learning features and classical radiomics features based on single-source dual-energy CT imaging	Osteoporosis and Osteopenia	MRI radiomics + demographics	424	Internal	182	0.98
Wang S. et al. (2024)	Combining deep learning and radiomics for automated, objective, comprehensive bone mineral density assessment from low-dose chest computed tomography	Lumbar Spine Osteoporosis	LDCT radiomics	239	External	100	0.94
Xie et al. (2022)	Development and validation of a machine learning-derived radiomics model for diagnosis of osteoporosis and osteopenia using quantitative computed tomography	Osteoporosis vs. Osteopenia	QCT radiomics + demographics + BMI + laboratory biomarkers^2^	414	Internal	176	0.96
Zhang et al. (2024a)	Development and validation of a feature-based broad-learning system for opportunistic osteoporosis screening using lumbar spine radiographs	Osteoporosis	XR radiomics	1180	Internal	145	0.80
Zhen et al. (2024)	Comparative evaluation of multiparametric lumbar MRI radiomic models for detecting osteoporosis	Osteoporosis	MRI radiomics	112	External	35	0.82
Zhao et al. (2022)	Fully automated radiomic screening pipeline for osteoporosis and abnormal bone density with a deep learning-based segmentation using a short lumbar mDixon sequence	Osteoporosis and Abnormal BMD	MRI radiomics + demographics + BMI	142	External	25	0.91

^#^ External validation when available, otherwise internal.

1 Blood pressure, Triglycerides, Hemoglobin, Fasting blood glucose, and Total cholesterol.

2 Hemoglobin, Glucose, Total bilirubin, Direct bilirubin, Indirect bilirubin, Alkaline phosphate, Uric acid, Calcium, Magnesium, Phosphate, Homocysteine.

AUROC: Area under the Receiver Operating Characteristic Curve. BMD: Bone Mineral Density. Body Mass Index (BMI): calculated as weight (kilograms) divided by the square of the height (meters). CNNs: Convolutional neural networks. CT: Computed Tomography. DECT: Dual-energy Computed Tomography. Demographics: (Age, Sex). LDCT: Low-dose Computed Tomography. MRI: Magnetic Resonance Imaging. QCT: Quantitative Computed Tomography. VA: Vertebral Augmentation. VCFs: Vertebral Compression Fracture. XR: X-Ray.

### 3.3 Prediction of fragility vertebral fractures

Osteoporotic vertebral compression fractures (OVCFs), the hallmark of osteoporosis (Grigoryan et al., 2003), affect approximately one in three women and one in five men over 50 years (Ballane et al., 2017) (Table 3). OVCFs are associated with increased morbidity, chronic pain, spinal deformity, decreased physical function, and elevated mortality risk (Grigoryan et al., 2003; Kado et al., 1999). With OVCF often asymptomatic or presenting with nonspecific back pain (Fink et al., 2005), research suggests that approximately 2/3 may not come to clinical attention (Gehlbach et al., 2000). Current standard clinical OVCF assessment employs visual evaluation of quantitative morphometry on lateral radiographs (Grigoryan et al., 2003; Black et al., 1991) or via vertebral fracture assessment (VFA) from DEXA imaging (Lewiecki and Laster, 2006; Zeytinoglu et al., 2017). Despite widespread adoption and established reliability (κ = 0.74) (Bae et al., 2025; Genant et al., 1993), both protocols are inherently subjective, requiring experienced readers to differentiate true OVCF from degenerative changes and congenital anomalies (Ferrar et al., 2005; Wang et al., 2020). Significantly, mild (grade 1) OVCFs are frequently missed in clinical practice (Wang et al., 2020; Delmas et al., 2005). Hence, radiographic diagnosis remains a defining challenge in OVCF management (Grigoryan et al., 2003). In a study evaluating T1-weighted MR imaging of 50 menopausal women with radiographic evidence of OVCF and 50 healthy women with no evidence of OVCF, Grey-level texture features, specifically, higher contrast and entropy radiomics measures, indicating higher heterogeneity of the bone architecture, achieved an AUC = 0.7 for discriminating OVCF based on ROC analysis (Zaworski et al., 2021). Yang et al. (2022) extracted first-order features and gray level texture features from CT, with model selection performed using LASSO in 147 patients having OVCF verified on CT and MR imaging. The CT-radiomics model showed an accuracy, sensitivity and specificity of 77.27, 66.67% and 84.62%, respectively, for distinguishing acute vs. chronic OVCFs, highlighting radiomics analysis potential for temporal fracture classification. A study of patients with OVCFs (n = 77) and non-OVCFs (n = 92), combining deep transfer learning with ResNet-50 and radiomic features (first-order, shape and gray level textural) derived from CT data showed the combined model to achieve a concordance index of 0.839 in the training set and 0.795 in testing set for risk of OVCF (Zhang et al., 2024b), which was significantly higher (p < 0.05) than the performance of a model based on clinical indicators only (Zhang et al., 2024b). Similar findings were reported for models derived from MR data employing a gradient boosting algorithm (XGBoost) to predict OVCF after vertebral augmentation (AUC: 0.90, sensitivity: 0.90, and specificity: 0.73) (Cai et al., 2023) and in patients at risk for imminent new vertebral fractures in patients with OVCFs undergoing vertebral augmentation (Jiang et al., 2023a; Liu et al., 2023), Figure 6. These studies illustrate the value of radiomics in providing predictive insights into fracture risk and treatment outcomes, potentially aiding clinical decision-making for high-risk patients.

**TABLE 3:** List of radiomics models for the prediction of fragility vertebral compression fracture models.

References	Title	Prediction target(s)	Model features	Training cohort	Mode of validation^#^	Validation cohort	Performance (AUC-ROC)
Cai et al. (2023)	MRI-based radiomics assessment of the imminent new vertebral fracture after vertebral augmentation	New vertebral fracture after VA	MRI radiomics + demographics + VCF status	126	Internal	42	0.90
Del Lama et al. (2022)	Computer-aided diagnosis of vertebral compression fractures using convolutional neural networks and radiomics	VCF L1-L5	MRI radiomics + CNN intermittent layers + demographics	55	Internal	6	0.98
Duan et al. (2023)	Differential diagnosis of benign and malignant vertebral compression fractures: Comparison and correlation of radiomics and deep learning frameworks based on spinal CT and clinical characteristics	Benign vs. malignant VCF	T radiomics + demographics + site of VCF (Thoracic vs. lumbar)	224	Internal	56	0.99
Jiang et al. (2023b)	Preoperative prediction of new vertebral fractures after vertebral augmentation with a radiomics nomogram	New vertebral fracture after VA	MRI radiomics + demographics + BMD + radiomics signature + IVC + clinical parameters^1^	153	External	44	0.798
Jiang et al. (2023a)	Development and validation of a machine learning model to predict imminent new vertebral fractures after vertebral augmentation	New vertebral fracture after VA	MRI radiomics + demographics + number of treated vertebrae + location of treated vertebrae + surgical procedures	138	External	38	0.907
Kim et al. (2022)	Prediction of the acuity of vertebral compression fractures on ct using radiologic and radiomic features	Acuity of VCF	CT radiomics + cortical disruption + cleft or line + radiomic score	122	External	32	0.95
Li W. G. et al. (2023)	The value of radiomics-based CT combined with machine learning in the diagnosis of occult vertebral fractures	Occult VF	CT radiomics	102	Internal	26	0.882
Liu et al. (2023)	Novel radiomics-clinical model for the noninvasive prediction of new fractures after vertebral augmentation	New fractures after VA	MRI radiomics + demographics + BMI + clinical parameters^2^	101	5-Fold Cross-Validation	NA	0.754
Nian et al. (2024)	Development and validation of a radiomics-based model for predicting osteoporosis in patients with lumbar compression fractures	Osteoporosis in patients with lumbar spinal fractures	MRI radiomics + demographics + BMI + clinical parameters^3^	102	Internal	26	0.84
Park et al. (2022)	Automated segmentation of the fractured vertebrae on CT and its applicability in a radiomics model to predict fracture malignancy	Fracture malignancy	CT radiomics	158	External	59	0.83
Saravi et al. (2024)	Integrating radiomics with clinical data for enhanced prediction of vertebral fracture risk	Vertebral fracture risk prediction	CT radiomics + demographics + BMD	104	External	20	0.89
Seol et al. (2024)	Predicting vertebral compression fracture prior to spinal SBRT using radiomics from planning CT	VCF	CT radiomics + demographics + radiotherapy parameters + VF occurrence + SINS	80	Internal	34	0.871
Wang M. et al. (2023)	A computed tomography-based radiomics nomogram for predicting osteoporotic vertebral fractures: a longitudinal study	Osteoporotic vertebral fracture	CT radiomics	216	10-Fold Cross-Validation	NA	0.87
Wang X. et al. (2023)	Value of (18)F-FDG-PET/CT radiomics combined with clinical variables in the differential diagnosis of malignant and benign vertebral compression fractures	Benign vs. malignant VCF	PET/CT radiomics + clinical parameters^4^	100	Internal	44	0.962
Wang J. et al. (2024)	Predicting secondary vertebral compression fracture after vertebral augmentation via CT-based machine learning radiomics-clinical model	Secondary VCF after VA (low risk vs. high risk)	CT radiomics + demographics + BMI + clinical parameters^5^ + BMD + VF number and location	329	Internal	141	0.884
Xue et al. (2022)	Using radiomic features of lumbar spine CT images to differentiate osteoporosis from normal bone density	Osteoporosis	CT radiomics	133	5-Fold Cross-Validation	NA	0.994
Yang et al. (2022)	Prediction of acute versus chronic osteoporotic vertebral fracture using a radiomics-clinical model on CT.	Acute vs. chronic VCF	CT radiomics + demographics + VCF	103	Internal	44	0.86
Yang J. et al. (2025)	CT-based radiomics predicts adjacent vertebral fracture after percutaneous vertebral augmentation	Adjacent VF after percutaneous VA	CT radiomics + demographics + BMI + hypertension + diabetes + VF history	110	Internal	48	0.86
Zhang J. et al. (2023)	Differentiation of acute and chronic vertebral compression fractures using conventional CT based on deep transfer learning features and hand-crafted radiomics features	Acute vs. chronic VCF	CT radiomics + demographics	416	Internal	104	0.946
Zhang et al. (2024b)	Development and validation of a predictive model for vertebral fracture risk in osteoporosis patients	Osteoporotic vertebral fracture	CT radiomics + demographics + BMI + BMD	135	Internal	34	0.971
Zhang et al. (2024c)	Exploring deep learning radiomics for classifying osteoporotic vertebral fractures in X-ray images	Osteoporotic VF (class 0–2 classification)	X-ray radiomics	712	External	111	0.940
Zhang et al. (2024d)	Constructing a deep learning radiomics model based on X-ray images and clinical data for predicting and distinguishing acute and chronic osteoporotic vertebral fractures: a multicenter study	Acute vs. chronic osteoporotic VF	X-ray radiomics + demographics:+ DEXA T-scores	712	External	111	0.895
Zhuang et al. (2025)	Diagnosis of acute versus chronic thoracolumbar vertebral compression fractures using CT radiomics based on machine learning: a preliminary study	Acute vs. chronic thoracolumbar VCF	CT radiomics + demographics + chief complaint (1. trauma; 2. pain; 3. others)	136	External	41	0.875

^#^ External validation when available, otherwise internal.

1 Surgical procedure, number of treated vertebrae, location of treated vertebrae, number of previous VF.

2 BMD, cause of OVCF, PKP, or PVP, augmentation method, unilateral or bilateral puncture method, DEXA, results.

3 Height, Weight, Ca2+, WBC, CRP, ESR, DEXA, results, T-lumbar, T-femoral neck, T-hip, T-sum, T-lowest, Z-score.

4 SULpeak, SULmax, SUVpeak, SUVmax, Age, Osteolytic destruction, Fracture line, Soft tissue mass or swelling, Appendices or posterior vertebrae involvement.

5 SVCF, status, Height, Weight, Smoking status, Alcohol consumption, Bone cement use, Cement leakage, T-score of L1-L4, Average T-score of L1-L4, T-score of W.

AUROC: Area under the Receiver Operating Characteristic Curve. Body Mass Index (BMI): calculated as weight (kilograms) divided by the square of the height (meters). Demographics: (Age, Sex). DEXA: Dual-Energy X-Ray Absorptiometry. SINS: Spine Instability Neoplastic Score. VA: Vertebral Augmentation. VCF: Vertebral Column Fracture. VF: vertebral fracture.

**FIGURE 6:**
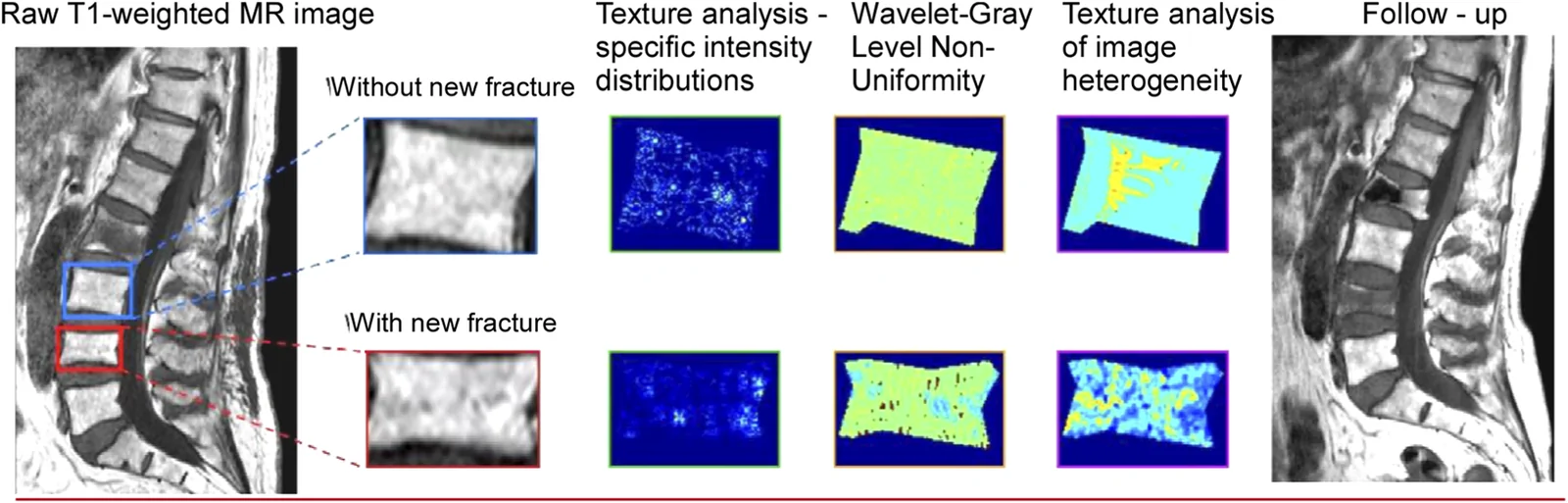
An example of radiomics feature maps for identifying vertebral bodies that did or did not fracture. For each of the features, the vertebral body with a new fracture (Lower) shows higher heterogeneity than the non-fractured vertebra (Upper), the differences found to be significant (Liu et al., 2023). Image adapted from Liu et al., Acad Radiol 2023; 30:1092–1100 (Liu et al., 2023).

### 3.4 Chronic low back pain (LBP)

LBP is a clinical entity with a mean ± SD lifetime prevalence of 38.9% ± 24.3% (Hoy et al., 2014) (Table 4). LBP forms one of the leading causes of disability in the adult population and imposes a significant medical, economic, and social burden worldwide. Intervertebral disc degenerative disease (DDD) is a radiographic-anatomical finding (Hooten and Cohen, 2015) characterized by alterations to the disc’s tissue biochemical composition and structure under physiological and pathological stresses (Sneag and Potter, 2017). MRI, the gold standard noninvasive method for assessing DDD in patients, allows for the detection of anatomical changes, collagen degradation, proteoglycan depletion, and other potential pain-generating defects of the IVD (Paul et al., 2018). Although patients with LBP can present DDD on MRI, DDD is frequently found in imaging studies of asymptomatic patients. Effectively interpreting these MR images when required to establish whether there is a causative relationship between LBP and DDD findings on MRI remains a critical challenge for treating clinicians.

**TABLE 4:** List of radiomics models for assessment of intervertebral disc pathology.

References	Title	Prediction target(s)	Model features	Training cohort	Mode of validation^#^	Validation cohort	Performance (AUC-ROC)
Fan et al. (2024)	Deep-learning-based radiomics to predict surgical risk factors for lumbar disc herniation in young patients	Surgical risk factors in young LDH patients	MRI radiomics + Demographics + clinical parameters^1^	1,257	External	191	0.941
Lin A. et al. (2024)	Radiomics based on MRI to predict recurrent L4-5 disc herniation after percutaneous endoscopic lumbar discectomy.	Recurrent disc herniation after PELD	MRI radiomics	146	3-fold cross-validation	341	0.86
McSweeney et al. (2025)	Robust radiomic signatures of intervertebral disc degeneration from MRI	Pfirrmann grade classification	MRI radiomics	5,588	5-fold cross-validation	1397	0.94
Saravi et al. (2024)	Integrating radiomics with clinical data for enhanced prediction of vertebral fracture risk	Vertebral fracture risk prediction	CT radiomics + Demographics + BMD + number of segmented vertebrae + number of fractures	124	External	20	0.89
Saravi et al. (2023)	Clinical and radiomics feature-based outcome analysis in lumbar disc herniation surgery	Surgical outcomes (complications, LOS, operation time)	MRI radiomics + BMI + clinical parameters^2^	172	5-fold cross-validation	NA	0.92
Tkachev (2024)	The power of radiomics: machine learning predicts MRI features of resorption of the lumbar disc herniation	Disc herniation resorption rate	MRI radiomics	171	Internal	NA	0.83
Xie et al. (2024)	MRI radiomics-based decision support tool for a personalized classification of cervical disc degeneration: a two-center study	Cervical disc degeneration classification (Pfirrmann grade)	MRI radiomics	305	Internal	130	0.95
Waldenberg et al. (2023)	Associations between vertebral localized contrast changes and adjacent annular fissures in patients with low back pain: a radiomics approach	Outer Annular Fissure in an Adjacent Intervertebral Disc	MRI radiomics + clinical parameters^3^	61	5-fold cross-validation	NA	0.76
Wang et al. (2026)	Development and validation of a prognostic prediction model for lumbar-disc herniation based on machine learning and fusion of clinical text data and radiomic features	Early recurrence after PELD	MRI radiomics	300	Internal	128	0.76

^#^ External validation when available, otherwise internal.

1 BMI, occupational physical activity, Smoking status, Alcohol status, Recreational drug usage, Education, Income, Diabetes, Hypertension, Adverse psychosocial history, Depression score, Anxiety score, VAS, score, ODI, score, Lumbar lordosis angle, Lumbosacral angle, Sacral slope angle, Psoas major muscle CSA, Quadratus lumborum muscle CSA, Multifidus muscle CSA, Erector spinae muscle CSA, Subcutaneous fat tissue thickness at L1-L2 level, Low back pain, Sciatica, Prior conservative treatment, Superficial sensation abnormality, Abnormal reflex, Weakened muscle strength, SLR, test result, Michigan State University classification, Pfirrmann grade of disc degeneration, Modic change, Annulus fibrosus tear, Lumbodorsal myofascitis, Psoas major muscle fat infiltration, Quadratus lumborum muscle fat infiltration, Erector spinae muscle fat infiltration, Multifidus muscle fat infiltration.

2 Nicotine usage, Alcohol usage, Private vs. non-private insurance, ASA, score, Preoperative CRP, length of stay, Operation time, Surgeon Years of Experience with case surgery type, Number of surgeries with case surgery type at time of surgery.

3 Smoking history, Alcohol consumption history, Hypertension history, Diabetes mellitus history, CAD, history, VTE, history, Varicose veins history, NSAID, usage history, Duration of diseases, Manual labor history, Surgical segments, Modic changes, Muscle strength, Abnormal sensation, D-dimer, FDPs, Hepatitis B history, Lipid levels, Preoperative platelets, Preoperative total protein, Preoperative ALB, blood calcium, Blood potassium, Blood phosphorus, Surgical plan, Duration of surgery, Intraoperative bleeding, Hemostatic material use, Postoperative platelets, Postoperative total protein, Postoperative ALB, postoperative anticoagulant therapy.

AUROC: Area under the Receiver Operating Characteristic Curve. Body Mass Index (BMI): calculated as weight (kilograms) divided by the square of the height (meters). CT: Computed Tomography. Demographics: (Age, Sex, BMI). LDH: Lumbar Disc Herniation. LOS: Length of Stay. MRI: Magnetic Resonance Imaging. PELD: percutaneous endoscopic lumbar discectomy.

#### 3.4.1 Assessment of IVD degeneration

**FIGURE 7:**
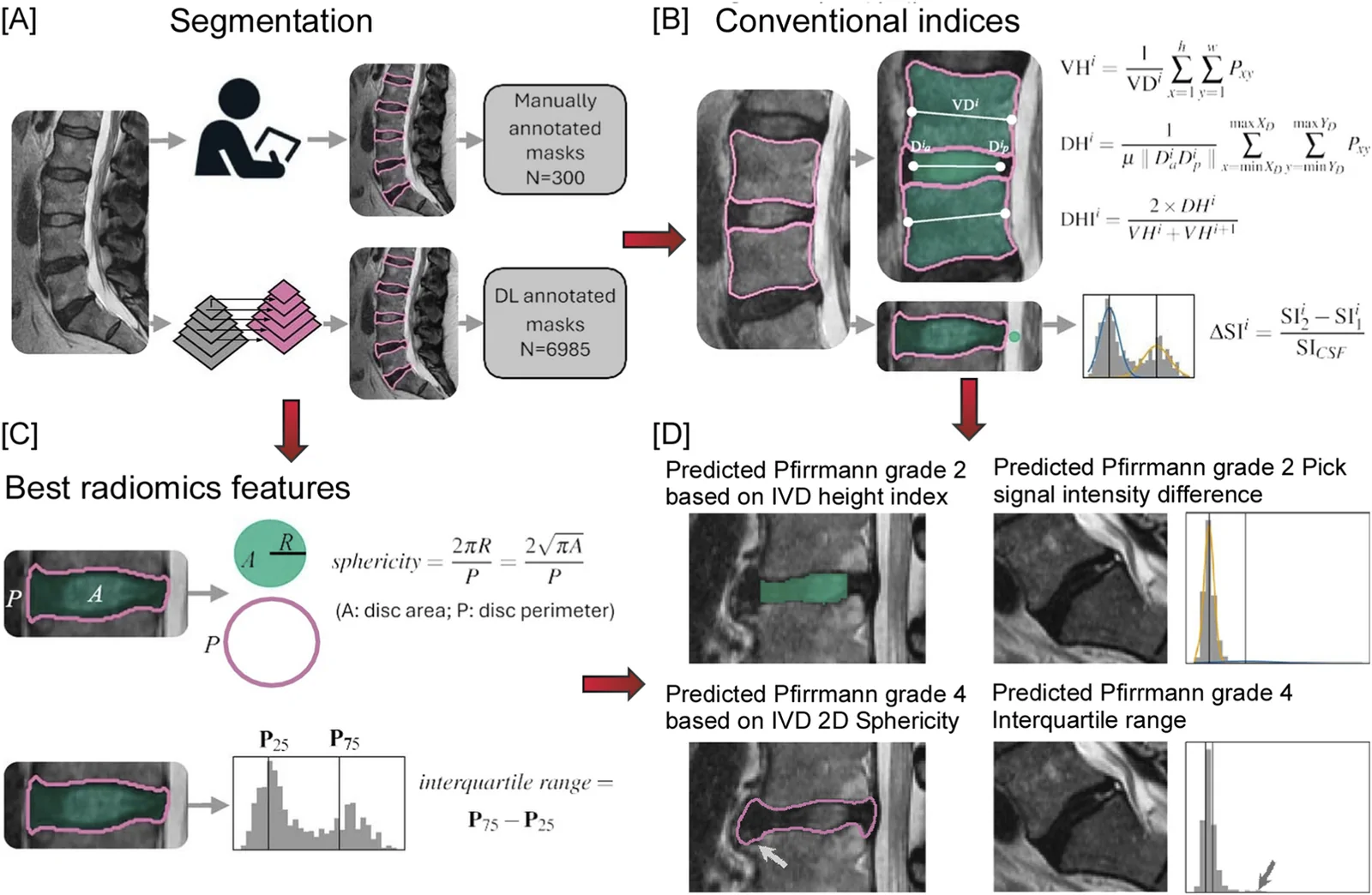
Manual and deep learning (DL) segmentation of the IVD and vertebral body **(A)** is processed for calculation of image-based indices per IVD **(B)** and to derive best-performing radiomic features **(C)** based on radiomic feature calculation and robustness analysis. Radiomics- based sphericity measure (capturing a combination of loss of IVD height, osteophyte formation, and vertebral endplate disruption) and interquartile range (reducing the influence of localized areas of high signal intensity in degenerated IVD on peak signal intensity difference) showed higher association with Pfirrmann grade than conventional indices of IVD height and peak signal intensity difference derived from DL segmentation **(D)** (McSweeney et al., 2025). Adapted from McSweeney et al. (2025) Spine, 2025 50(24):1737–1746.

Using 924 shape and texture features derived from T1WI and T2WI MR data of cervical discs, Xie et al. (2024) evaluated radiomics model performance for predicting discs’ Pfirrmann IVD degradation-based grading (Pfirrmann et al., 2001). The analysis found features indicating higher-order textural heterogeneity to contribute 80% of the model’s discriminatory power. First-order (40%) and textural grey-level features (20%) were dominant factor types for cervical disc degeneration classification. Kurtosis features appeared frequently among top-ranked parameters for both T1W data, with the T2WI-based radiomics model outperforming the T1-based model for disc grading. McSweeney et al. (2025) extracted first-order statistics, textural and shape radiomic features, as well as conventional geometrical and intensity measures from T2WI MR images of lumbar discs (Figure 7). The study found the radiomics model to outperform the model based on conventional indices, with 2D sphericity and interquartile range emerging as robust signatures highly correlated with Pfirrmann grade (Spearman’s ρ = −0.72 and −0.77, respectively). Using a multilayer-perceptron DL model for model selection, reducing 174 histogram- and texture-based MR-radiomic features to three key vertebral marrow texture descriptors, the radiomics model achieved 83% accuracy, 97% sensitivity, 28% specificity in distinguishing levels neighboring painful annular fissures (discography-proven) from intact discs in 61 chronic LBP patients who underwent conventional MRI followed by CT-discography (Waldenberg et al., 2023).

#### 3.4.2 IVD herniation detection and classification

Lumbar disc herniation is a ubiquitous condition diagnosed in patients with LBP (Schroeder et al., 2016). Saravi et al. (2024) combined clinical outcomes (hospital length of stay [LOS], surgery technique, ASA physical status classification, demographic information, and preoperative C-reactive protein) with MR radiomics features [first-order statistics, shape-based (2D and 3D) and grey-level texture] extracted from T2WI MR data of 81 women and 91 male patients with Lumbar disc herniation who had undergone either a microsurgical or full-endoscopic procedure. The study identified gray-level co-occurrence matrix (GLCM), first-order statistics, and neighboring gray-tone difference matrix (NGTDM) features as most predictive of surgical outcomes, including complications, LOS, and operation time. However, the combined radiomics and clinical feature-based model (mean accuracy of 88.2% ± 2.6% in testing cohort) provided little improvement compared to a clinical features model (87.69% ± 3.62%), questioning the utility of inclusion of the radiomics features for improvement in predictive capacity (Saravi et al., 2023). Similar findings were reported by Fan et al. (2024), comparing MR-radiomics (T2WI: shape, intensity, and texture-based features) with model selection based on 1) a machine learning approach, 2) a DL model trained on imaging features, and 3) a DL radiomics nomogram (DLRN) integrating clinical parameters [Oswestry Disability Index (ODI), Pfirrmann grade, straight leg raise test (SLRT), modified Macnab functional index (MMFI), and MSU classification of herniation morphology] for predicting LDH. Evaluated in a retrospective cohort of 1066 patients aged 16–44 with LBP, the DLRN model performed significantly better than the MR-radiomics or DL model alone, suggesting clinical information not captured by imaging, such as pain, status of musculature and neurological status, has an important role in predicting LDH.

Spontaneous resorption of herniated disc material occurs in approximately 66%–76% of patients, with disc herniation (Zhong et al., 2017). Predicting which patients will experience resorption could guide conservative versus surgical management decisions. In a preliminary study of 171 patients, a machine learning model based on MR-radiomics texture features had an overall accuracy of 83% in differentiating between fast, medium, slow, and prolonged resorption groups (Tkachev, 2024). This study found the extracted textural features to be significant biomarkers for changes in herniated disc structure, suggesting the utility of radiomics models for quantifying enhanced microcirculation and tissue nourishment in the herniated anatomy of the disc.

In patients treated with surgery for LDH, incidence rates for recurrent lumbar disc herniation (rLDH) range from 2.8% to 15% (Zhou et al., 2018). Evaluating this risk to inform perioperative, individualized management of LDH patients remains an unmet clinical need. In a study of 167 patients who developed rLDH out of 487 patients who underwent percutaneous endoscopic lumbar discectomy (PELD), Lin A. et al. (2024) screened 1,040 MRI-based (T2WI) radiomic features (first-order statistics, 2D and 3D shape-based, gray-level matrix, and wavelet-based), demonstrating 18 features (3 first-order, 2 shape-based and 13 texture grey-level based) that effectively predicted rLDH risk in PELD-treated patients. Evaluated in 428 patients (196 males, 232 females) treated using PELD, Wang et al. (2026) found a model combining machine learning based radiomics features selection with clinical indicators to strongly predict rLDH (AUC = 0.820 in the test set), supporting the utility of MR-radiomics models for improving the prediction of rLDH beyond that of traditional prognostic clinical-profile features. The studies reviewed highlight that MR baseline radiomic signatures may offer prognostic value to identify patients at high risk for accelerated disc degeneration, thus enabling early intervention strategies. Significantly, the relationship between specific radiomic patterns reflecting increased disc tissue heterogeneity and structural disorganization may indicate cellular senescence, inflammatory activation, and matrix degradation, all of which drive degeneration progression in the disc (Ngo et al., 2017; Vo et al., 2016).

#### 3.4.3 Current limitations

However, disc-based radiomics studies face several limitations. Differences in the MR scanner and scanner-specific image acquisition variations, MR experimental parameters and the reconstruction algorithms require data harmonization efforts, including standardized protocols and computational normalization methods across differences in MR scanners and institutions, which are essential for multi-center research and clinical deployment. Disc tissue segmentation on clinical MR data offers a unique challenge that significantly influences radiomic analysis outcomes. Manual segmentation by experts remains the standard. However, defining disc anatomy and that of its tissue is time-consuming and subject to inter- and intra-observer variability. Semi-automatic segmentation algorithms, such as region-growing (Saravi et al., 2023) and DL based (Xie et al., 2024) approaches, may potentially improve efficiency and consistency.

Combined with inadequate description and standardization of image segmentation of disc tissue, these limitations potentially limit feature extraction or model building methodology (Buvat and Orlhac, 2019; Papanikolaou et al., 2020), all of which substantially impact feature values (Kocak et al., 2023), radiomics model development and generalizability, representing a critical challenge for clinical translation.

### 3.5 Cancer-mediated metastatic spine disease

With cancer therapy extending patients’ life expectancy and improving cancer prognosis (Wewel and O'Toole, 2020) (Table 5), the incidence of metastatic spine disease (MSD) (Morimoto et al., 2024) continues to increase (Van den Brande et al., 2022). Spinal bone metastases can lead to devastating complications, including vertebral fractures, estimated to affect up to 16% (Van den Brande et al., 2022) of the 5.4 million cancer patients with MSD in the US (2022) (U.S. Cancer Statistics Working Group et al., 2022), spinal instability, severe pain and metastatic epidural spinal cord compression (MESCC) (Van den Brande et al., 2022; Amelot et al., 2024), a severe neurological complication, all of which impact patient quality of life and survival (Oster et al., 2013; Saad et al., 2007). Clinical diagnostics relies on qualitative visual interpretation and subjective measurements, which fail to capture the full complexity of tumor heterogeneity and biomechanical compromise (Confavreux et al., 2021), profoundly impacting patients’ clinical care (Confavreux et al., 2021; Yao et al., 2017). In the metastatic spine, radiomics analysis has been applied to several areas of diagnostic/research.

**TABLE 5:** Radiomics for spinal metastasis.

References	Title	Prediction target(S)	Model features	Training cohort	Mode of validation^#^	Validation cohort	Performance (AUC-ROC)
Differentiation of benign versus malignant lesions							
Cao et al. (2024)	Radiomics model based on MRI to differentiate spinal multiple myeloma from metastases: A two-center study	Spinal multiple myeloma vs. BM	MRI radiomics	210	External	53	0.87
Chen et al. (2022)	Differentiation between spinal multiple myeloma and metastases originated from lung using multi-view attention-guided network	Multiple myeloma vs. lung-originated BM	MRI radiomics	217	5-fold cross-validation	NA	0.78
Chianca et al. (2021)	Radiomic machine learning classifiers in spine bone tumors: a multi-software, multi-scanner study	Spine BM	MRI radiomics	100	External	35	0.83
Gitto et al. (2022)	Diffusion-weighted MRI radiomics of spine bone tumors: feature stability and machine learning-based classification performance	Benign vs. malignant BM	MRI radiomics	101	10-fold cross-validation	NA	0.78
Hinzpeter et al. (2022)	Radiomics for detecting prostate cancer bone metastases invisible in CT: a proof-of-concept study	BM	CT radiomics	328	External	NA	0.90
Jin et al. (2022)	Application of 18F-FDG PET-CT images based radiomics in identifying vertebral multiple myeloma and bone metastases	Multiple myeloma vs. BM	MRI radiomics + CT radiomics	263	10-fold cross-validation	NA	0.97
Li S. et al. (2023)	MRI-based radiomics nomogram for differentiation of solitary metastasis and solitary primary tumor in the spine	Solitary BM vs. Solitary primary tumor	MRI radiomics	98	Internal	37	0.92
Liu et al. (2022)	Vertebral MRI-based radiomics model to differentiate multiple myeloma from metastases: influence of features number on logistic regression model performance	Multiple myeloma vs. BM	MRI radiomics + patient + tumor parameters	241	5-fold cross validation	NA	0.85
Mesguich et al. (2021)	Improved 18-FDG PET/CT diagnosis of multiple myeloma diffuse disease by radiomics analysis	Multiple myeloma	($,*, %,@)	20	Internal	10	0.80
Naseri et al. (2022)	Radiomics-based machine learning models to distinguish between metastatic and healthy bone using lesion-center-based geometric regions of interest	BM in lung cancer patients	PET-CT radiomics	359	5-fold cross-validation	NA	0.90
Naseri et al. (2023)	A scalable radiomics- and natural language processing-based machine learning pipeline to distinguish between painful and painless thoracic spinal bone metastases: retrospective algorithm development and validation study	painful vs. painless BM	CT radiomics	121	Internal	55	0.83
Sanli et al. (2022)	Radiomics biopsy signature for predicting survival in patients with spinal bone metastases (SBMs)	Six-month survival for patients with spinal BM	CT radiomics	150	Internal	100	0.69
Zhang S. et al. (2023)	An MRI-based radiomics nomogram for differentiating spinal metastases from multiple myeloma	Spinal multiple myeloma vs. BM	CT radiomics + demographics + clinical parameters^1^	146	External	101	Zhang S. et al. (2023)
Zheng F. et al. (2024)	Comparison of different fusion radiomics for predicting benign and malignant sacral tumors: a pilot study	Benign vs. malignant sacral tumors	MRI radiomics + demographics + tumor parameters ($, @, #, *, &)	107	Internal	27	0.96
Zhu et al. (2025)	Radiomics analysis of thoracic vertebral bone marrow microenvironment changes before bone metastasis of breast cancer based on chest CT	BM	MRI radiomics	84	Internal	22	0.93
Prediction of Malignant Vertebral Compression Fracture (MVCF)							
Chee et al. (2021)	Combined radiomics-clinical model to predict malignancy of vertebral compression fractures on CT	Malignancy of VCFs	MRI radiomics	110	Internal	55	0.948
Chiari-Correia et al. (2023)	A 3D radiomics-based artificial neural network model for benign versus malignant vertebral compression fracture classification in MRI	Benign vs. malignant VCF	CT radiomics + age + history of malignancy	61	Internal	30	0.98
Duan et al. (2023)	Differential diagnosis of benign and malignant vertebral compression fractures: Comparison and correlation of radiomics and deep learning frameworks based on spinal CT and clinical characteristics	Osteoporotic vs. malignant VCF	CT radiomics	280	Internal	56	0.97
Feng et al. (2024)	An MRI-based radiomics nomogram for differentiation of benign and malignant vertebral compression fracture	Benign vs. malignant VCF	MRI radiomics + demographics + tumor involvement parameters^2^	113	Internal	56	0.90
Frighetto-Pereira et al. (2016)	Shape, texture and statistical features for classification of benign and malignant vertebral compression fractures in magnetic resonance image.	Osteoporotic vs. malignant VCF	MRI radiomics	63	10-Fold Cross-Validation	NA	0.92
Geng et al. (2024)	Radiomics based on multimodal magnetic resonance imaging for the differential diagnosis of benign and malignant vertebral compression fractures	Benign vs. malignant VCF	MRI radiomics + demographics + malignancy and disease progress	172	Internal	38	0.982
Gui et al. (2022)	Radiomic modeling to predict risk of vertebral compression fracture after stereotactic body radiation therapy for spinal metastases	VCF after SB radiation therapy	MRI radiomics	95	Leave-One-Out Cross Validation	NA	0.878
Zhang H. et al. (2023)	Differentiation of benign versus malignant indistinguishable vertebral compression fractures by different machine learning with MRI-based radiomic features	Benign vs. malignant VCFs	MRI radiomics	263	External	103	0.84

^#^ External validation when available, otherwise internal.

1 Primary tumor type metastasis, Location treated spinal metastases causing symptoms, Radiation field, Radiotherapy fractionation schedule, Pathological fracture, Spinal compression, Lymphatic metastases, Pain score, Visceral metastases, Brain metastases, World Health Organization performance score.

2 Convex posterior vertebral border, Pedicle or posterior element involvement, Epidural mass, Paraspinal mass, Retropulsion of a posterior bone fragment, Low signal intensity band, Fluid sign, Spared normal marrow signal.

AUROC: Area under the Receiver Operating Characteristic Curve. BM: vertebral bone metastasis. Body Mass Index (BMI): calculated as weight (kilograms) divided by the square of the height (meters). LASSO: least absolute shrinkage and selection operator. Demographics: (Age, Sex). SB: Stereotactic Body. Tumor: [$: Diameter, @: Margin (well-defined/ill-defined), #: Heterogeneous appearance (present/absent), *: Shape (regular/irregular), &: Number of lesions (single/multiple), and %: signal].

#### 3.5.1 Differentiation of benign versus malignant lesions

Distinguishing benign vs. malignant bone lesions is essential for appropriate treatment planning, as management strategies differ substantially between these entities. Spinal anatomy complexity, the variability of lesion appearance, and the effect of age on overlapping features between benign and malignant lesions pose significant challenges for differentiating malignant from benign spinal lesions on CT (Sciubba et al., 2010). Applied to MRI data, radiomics-based models derived from shape-, first-order-, and texture-based features demonstrated high performance for differentiating spinal multiple myeloma (MM) lesions from vertebral BM (Hsieh et al., 2023; Jin et al., 2022). In a two-center study in which 263 multiple myeloma patients were used for the training cohort (Center 1), with Center 2 providing an external validation cohort, Cao et al. (2024) showed that radiomic features extracted from contrast-enhanced T1WI (CET1) and T2WI MR data demonstrated 0.87 accuracy for differentiating spinal MM lesions from bone metastasis (BM). Radiomics models higher performance for differentiating benign versus malignant lesions compared to models based on clinical features alone, were similarly reported for diagnosis of diffuse MM disease based metabolic features in 18-FDG PET/CT imaging (Outcomes: measures of Standardized Uptake Value (SUV) and metabolic heterogeneity) (Mesguich et al., 2021), for differentiating solitary metastasis and solitary primary tumor in MM (Li S. et al., 2023) and, based on radiomics shape features, in lung cancer patients (Naseri et al., 2022). Zhang S. et al. (2023) evaluated the radiomics measures’ ability to quantify the lesion heterogeneity of shape (showing higher variation) and MR signal (higher in BM vs. MM based on textural measures), concluding that the radiomics models could achieve significantly higher differentiation than that of a model based on clinical features.

Increasingly, radiomics studies are using DL models for radiomics model selection. Fusing T2W MR imaging and non-contrast computed tomography (NCCT) in a cohort of 134 patients with pathologically confirmed sacral tumors, Zheng F. et al. (2024) demonstrated that the application of DL models (DenseNet121, ResNet50 and SimpleViT) for radiomics model selection was superior to statistical selection-based models (LASSO) for differentiating between benign and malignant sacral tumors This study highlighted the importance of complementary information from CT [quantification of matrix mineralization, structural integrity of the bone (Bailey et al., 2020)] and the MR [soft-tissue contrast resolution, anatomical mapping of the tumor’s extent within the bone and adjacent soft tissues (Sambri et al., 2022)], for delineating the tumors aggressiveness (Lodwick et al., 1980) based on features such as periosteal reaction, irregular osteolysis, or the presence of a soft-tissue mass and identification of benign vs. malignant margins of a bone tumor. Naseri et al. (Naseri et al., 2023) combined CT-based radiomics features (shape, intensity, texture) with a natural language machine learning pipeline for processing of clinical records to classify thoracic spinal metastases pain status, i.e., painful versus painless, in a cohort of 166 cancer patients (121 for training/validation and 55 for testing, with clinical notes matching ≤10 days post-CT). Although the model showed high performance for the training set (AUC = 0.940), it demonstrated degraded performance for the test set (AUC = 0.825). The authors credited the lower performance to the test set ratio of 1:5 between “pain” cases and “no pain” cases, highlighting the critical role of balance in the test sample to avoid high specificity (ability to identify pain instances properly) and low sensitivity (ability to identify no pain cases correctly). The study further demonstrated the importance of the selection of the lesion segmentation strategy due to the influence of vertebral anatomy and the ability of the model to extract image characteristics from outside the BM lesion.

#### 3.5.2 Diagnostic classification of benign vs. malignant vertebral fractures

Distinguishing malignant vertebral compression fractures (MVCFs) from osteoporotic (OVCFs) is a critical diagnostic challenge. A recent review (Zheng J. et al., 2024) found diagnostic accuracy to be significantly dependent on the imaging modality. Models based on extracting radiomics features from X-ray had the worst performance, attributed to the inherent deficiency of X-ray to identify tumor invasion adequately. MRI-based radiomics models had 89% sensitivity (95% CI: 86%–92%) and 88% (95% CI: 85%–91%) specificity in differentiating OVCFs from MVCFs (Li et al., 2019). Frighetto-Pereira et al. (2016) demonstrated an MR radiomics model incorporating statistical measures of gray levels, texture features, and shape descriptors of the vertebral body, including Fourier descriptors, to achieve high diagnostic performance (AUC = 0.92). Although MR images are considered the standard diagnostic modality for determining the benign or malignant nature of VCFs, some patients cannot be imaged due to health conditions, contraindications such as metal implants or their inability to pay for an MR examination. CT imaging provides detailed morphological information on bone integrity and fracture features such as the fracture line, vacuum phenomenon, and soft tissue mass, vital in the differential diagnosis of MVCFs vs. OVCFs (Schwaiger et al., 2016). However, this task is made difficult in elderly patients who may have combined osteoporosis and primary tumors from other organs, resulting in CT-based models being dependent on the radiologist’s experience and generally performing inferiorly to MR-based models (Li et al., 2018). Integrating DL convolutional neural network with traditional radiomic features, Duan et al. (2023) showed enhanced diagnostic performance (accuracy (ACC) of 0.93 and AUC of 0.97 on the validation set vs. (ACC: 0.91, AUC: 0.93) compared to DL model (ACC: 0.88, AUC: 0.89) alone in a study of 280 patients (155 with OVCFs and 125 with MVCFs). The authors suggested that the radiomics model evaluation of gray levels inhomogeneity of the fatty marrow attenuation and edema in OVCF, compared to lower intensity entropy and higher intensity skewness due to tumor infiltration and bone destruction in MVCFs than in OVCFs, leads to improved performance in the combined model (or models). Consistent with the clinical literature, the radiomics studies suggest that visual features such as the presence of a soft tissue mass and bone destruction were highly indicative of malignancy, while the existence of a transverse fracture line is indicative of a benign fracture (Kubota et al., 2005; Mauch et al., 2018). This study (Duan et al., 2023) further highlighted radiomics features with superior discriminative capabilities compared to the DL model alone, and their utility to enhance the interpretability of the DL models’ “black box.” The authors found a low correlation between the DL and radiomics features. The authors suggested that the DL model’s ability to interrogate paravertebral soft tissue was complementary to the radiomics feature concentration at the vertebrae, yielding the enhanced performance of the combined model. These studies highlight the potential of a combined radiomics and DL approach to aid in the differential diagnosis of benign and malignant vertebral fractures, thus reducing the need for confirmatory invasive diagnostic procedures, such as biopsies, enhancing the accuracy and efficiency of VCF diagnosis and treatment planning.

### 3.6 Limitations in radiomics-based models for spine research

Despite the promising applications of radiomics in predicting osteoporosis, differentiating benign versus malignant lesions and spinal fractures, several notable limitations impact the robustness, generalizability, and clinical utility of these models. Addressing these limitations is essential to advancing the field and ensuring the successful integration of radiomics into clinical workflows.

Across the included spine radiomics studies, the evidence base remains methodologically heterogeneous and largely exploratory. Most studies were retrospective, single-center, and modest in size, with variable segmentation strategies, preprocessing pipelines, feature selection methods, validation designs, and reporting of calibration or decision-curve metrics. Although many models report high AUC values, these values should be interpreted cautiously because small datasets, class imbalance, internal-only validation, and selective reporting can overestimate clinical performance. CT-based models generally showed more consistent feature stability due to more standardized intensity values, whereas MRI-based models offer superior soft-tissue and marrow contrast but are more vulnerable to scanner, sequence, and preprocessing variability. Deep learning and hybrid radiomics models may improve performance in some tasks, but they often reduce interpretability and require larger, externally validated datasets before clinical use.

Across the included clinical domains, the main limitation is not only whether radiomics can produce high-performing models, but whether these models provide reliable added value beyond established clinical imaging measures. In osteoporosis and vertebral fracture studies, radiomics may capture bone texture and microarchitectural heterogeneity beyond BMD, but many studies still use retrospective designs and limited validation cohorts. In intervertebral disc degeneration and low back pain, radiomics may quantify tissue heterogeneity, but multifactorial symptoms and a weak correlation between imaging findings and pain complicate clinical interpretation. In metastatic spine disease and benign versus malignant fracture differentiation, radiomics may improve lesion characterization, but model reliability depends on representative training data across tumor types, lesion patterns, and treatment states. These differences show that performance metrics should be interpreted within each clinical context rather than compared only by reported AUC.

#### 3.6.1 Sample size

Sample size is a common limitation across many radiomics studies. While many studies utilize a reasonable number of patients relative to their institutional resources, these sample sizes are often insufficient to represent the broader, diverse population. As discussed in a recent review by Riley et al. (2025), which also draws attention to Good Machine Learning guiding principles (U. S. Food and Drug Administration, 2021), an appropriate sample size is a crucial aspect for studies using prediction models. Insufficient sample size may lead to overfitting, where the model performs well on the specific training dataset but poorly on new, unseen data, thus restricting its generalizability. Small sample sizes also reduce the discrimination performance of the model, with the model not being able to distinguish well between the groups of interest. When considering overfitting, the researchers need to consider not only the sample size (n) but also the number of events. Maintaining a suitable ratio of outcomes with the predictor variables will stabilize the model and calibrate the predictions. Further on, for a machine learning model to generalize well (e.g., predict conditions like osteoporosis or fractures accurately), it needs to be trained and tested in the target population and setting with appropriate sample sizes, thus highlighting the need for larger datasets. Inadequate sample sizes also decrease the prediction uncertainty, i.e., the intervals around the prediction become too wide, thus decreasing the model’s utility. These aspects, as well as a more detailed overview of the sample size issues related to prediction models, can be found in the review from Riley et al. (2025) “Big data” cohorts, ideally gathered from multi-institutional or even international sources, are needed to capture the full variability in patient demographics and clinical presentations. Such data can help reduce overfitting and improve the model’s ability to generalize. However, collecting and managing these large datasets requires extensive resources and robust data-sharing protocols.

#### 3.6.2 Imaging parameters

The variability in image resolution and acquisition parameters across studies represents another major limitation. Minor differences in acquisition settings, such as voxel size, noise levels, or contrast adjustments, can lead to inconsistencies in extracted feature values. This issue is particularly pronounced in MR imaging, where inherent variability in factors like magnetic field strength, echo times, and scan protocols complicates reproducibility. For example, the Intraclass Correlation Coefficient (ICC), commonly used to measure inter-reader reliability (IRR) of radiomic features, often yields low reproducibility scores for MR imaging-derived features. This inconsistency is largely due to the inherent properties of MR rather than the radiomics methodology itself. As a result, many researchers recommend using computed tomography over MRI for radiomics in spine and orthopedic applications. CT imaging offers more standardized acquisition parameters and higher spatial resolution, resulting in greater feature stability and reliability, which is essential for clinical implementation.

#### 3.6.3 Standardized performance metrics

With the absence of standardized performance metrics for evaluating and comparing machine learning models across studies, studies employ a variety of performance metrics (detailed in Section 2.8), which complicates direct model comparison. This lack of standardization limits our ability to determine which models perform best for specific clinical applications. To address this, a meta-analysis approach has been suggested, where performance metrics from various studies could be aggregated and compared. However, such an approach requires the adoption of consistent metrics and reporting standards across studies. Establishing this standardization could involve consensus guidelines and benchmarks for reporting machine learning performance in radiomics, enabling more accurate assessments of model efficacy and promoting comparability across studies.

#### 3.6.4 Feature extraction techniques and model development pipelines

In addition to image acquisition inconsistencies, significant variability exists in feature extraction techniques and model development pipelines across radiomics studies. Differences in software platforms, preprocessing techniques, and segmentation protocols introduce inconsistencies that hinder reproducibility and reliability. These differences affect model performance, making it challenging to replicate results across institutions. Standardizing feature extraction protocols, as recommended by the Image Biomarker Standardization Initiative, and establishing consensus guidelines for preprocessing steps, imaging resolutions, and segmentation methods could help mitigate these challenges (Zwanenburg et al., 2020). Adopting such standards would allow for more reliable feature comparisons and facilitate multicenter studies. Along with IBSI-based feature definitions, standardized reporting should include acquisition parameters, preprocessing steps, segmentation methods, feature extraction software and version, feature selection strategy, validation design, calibration, and performance metrics.

#### 3.6.5 Clinical validation

Although radiomics models show promise in preliminary studies, few have undergone extensive clinical validation in real-world settings. This gap limits our understanding of how well these models can support clinical decision-making under diverse conditions. Additionally, few studies have explored how to integrate these models into existing clinical workflows effectively. For machine learning-based radiomics models to be clinically impactful, they must be user-friendly and compatible with electronic health record systems to support seamless clinical decision-making. To bridge this gap, collaboration among clinicians, radiologists, data scientists, and software developers is essential, along with prospective clinical trials to test model usability, reliability, and impact on patient outcomes in real-world settings. Across the studies summarized in this review, external validation remains limited; therefore, the reported performance metrics likely reflect model development performance more than real-world generalizability.

#### 3.6.6 Publication bias and selective reporting

Publication bias is an important limitation when interpreting the current spine radiomics literature. Studies reporting high AUC values, statistically significant radiomic signatures, and positive model performance are more likely to be submitted and published than studies showing poor, non-significant, or externally non-reproducible results. This may produce an inflated impression of model accuracy and clinical readiness. Selective reporting of the best-performing feature sets, algorithms, thresholds, or validation splits can further exaggerate performance, particularly when multiple models are tested, but only the strongest result is emphasized. This concern is especially relevant in radiomics, where high-dimensional feature extraction, small cohorts, class imbalance, and internal validation can generate optimistic estimates of performance.

Negative studies and failed external validations are also important because they identify unstable features, non-generalizable pipelines, and clinical scenarios where radiomics may add limited value beyond conventional imaging or clinical variables. Future studies should report all tested model types, validation strategies, and performance metrics, rather than only the highest-performing model. Where feasible, prospective registration of analysis plans, transparent reporting of excluded models, inclusion of calibration and decision-curve analyses, and publication of externally underperforming models would provide a more accurate assessment of the field. Greater transparency in reporting both positive and negative findings is needed to reduce evidence inflation and clarify which spine radiomics applications are most likely to translate into clinical use.

### 3.7 Future direction

Newer treatments, for example, targeted therapy and immunotherapy in cancer patients, target specific driver mutations and transmembrane receptors, respectively, with treatment efficacy significantly dependent on the identification of such biomarkers for selecting suitable treatment candidates. Traditionally, methods to identify such biomarkers have involved invasive focal tissue sampling, followed by methods such as Immunohistochemistry or molecular testing. However, such methods are invasive and may only examine a fraction of the disease or cancer process at a specific point in time and thus may face limitations in delineating the evolving disease process.

Multi-omics integrates multiple biological data types derived from several research fields, including: Molecular-omics (integrating transcriptomics, proteomics, genomics, metabolomics, and epigenomics that analyze molecular components, for example, DNA, RNA, proteins, metabolites, and epigenetic modifications), Pathological-omics (integrating digital pathology and histopathology data with molecular analysis), and Hematological-omics (analyzing the molecular and cellular components of blood cells underlying the biology of blood-related diseases), to analyze and understand biological systems comprehensively.

Radiomics’ remarkable ability to capture mesoscopic features, present in the imaging data but not readily observable to the human eye (Lu et al., 2019), may allow bridging of clinical imaging (“macroscopic”) with molecular imaging probes targeting specific biological processes. Such fusion may include the integration of radiomics with emerging technologies such as molecular imaging probes targeting specific biological processes (e.g., inflammation markers, matrix degradation enzymes), providing mechanistic insights linking underlying pathophysiology with imaging phenotypes (Ngo et al., 2017) and radiogenomics, correlating radiomic signatures with gene expression patterns, inflammatory marker profiles, and cellular characteristics (e.g., senescence markers) to elucidate biological mechanisms underlying imaging phenotypes (Busscher et al., 2010; Patil et al., 2019), yielding novel therapeutic targets and the development of new diagnostic protocols, allowing prophylactic disease-modifying interventions for intervertebral disc disease.

Such future developments are most evident in the cancer field. Integrated with machine learning, the application of radiomics enabled a more precise selection of immunotherapy candidates, improving patient stratification and treatment outcomes (Montoya et al., 2023), and the development of a non-invasive biomarker using tumoral and peritumoral CT-radiomics to differentiate responders to immune checkpoint inhibitors and predict patients’ survival outcomes (Wu et al., 2023).

- Liquid biopsies use serum biomarkers to facilitate precision medicine. Given the minimally invasive nature of both methods, liquid biopsy and radiomics can be both acquired and used alongside each other once their complementary and synergistic utilities are established. Prior studies have explored combining them with radiomics to improve their performance (Cucchiara et al., 2021). However, there are currently limited publications in this domain.
- Radiomics offers assessment at any time when appropriate imaging is acquired, and is non-invasive and low-cost. Delta radiomics targets the longitudinal variation in features resulting from evident changes in the disease or its management (Nardone et al., 2021). In patients treated with radiotherapy, delta radiomics was found to be associated with worse prognoses (Shi et al., 2020) and overall survival in patients with recurrent malignant glioma (Chang et al., 2019). In osteosarcoma patients, radiomics features to detect changes in tumor texture showed improved prediction after chemotherapy and/or radiotherapy for sarcoma (Lin et al., 2020). Although promising, limitations such as the dependency of the reliability of radiomics on the region of interest (ROI) examined (Brooks and Grigsby, 2014), therapy may significantly reduce or alter the post- vs. pre-therapy ROI, likely to affect the robustness of the measurement, as well as the lack of methodological studies addressing known limitations of radiomics analysis (Section 3.7).

Despite these limitations, radiomics analysis offers the possibility of becoming an optimal surrogate biomarker to test different treatment strategies and to develop a new generation of radiomics studies to investigate pathologic changes in spinal tissues non-invasively. Clinical translation will require a defined pathway from proof-of-concept modeling to prospective clinical testing. Future spine radiomics studies should be prospectively evaluated in multicenter cohorts, externally validated against predefined clinical endpoints, and tested for added value over standard imaging and clinical variables. Successful deployment will also require standardized acquisition, preprocessing, segmentation, and reporting frameworks; transparent model documentation; regulatory review as clinical decision-support software; cost-effectiveness analyses; and integration into PACS (Picture Archiving and Communication Systems) and EHR (Electronic Health Record) workflows so outputs can be reviewed by radiologists and treating clinicians without disrupting routine care. Future studies should also assess clinician acceptance, model failure modes, turnaround time, and whether radiomics-guided decisions improve patient outcomes.

A practical clinical translation pathway should proceed in stages. First, radiomics models should be developed using standardized imaging protocols, predefined segmentation methods, and transparent feature extraction pipelines. Second, models should be tested in temporally separated internal cohorts and then externally validated across independent institutions. Third, clinically useful models should be evaluated prospectively to determine whether they change diagnosis, risk stratification, treatment planning, or follow-up decisions compared with standard imaging alone. Fourth, implementation studies should assess whether radiomics outputs can be integrated into radiology reports, PACS viewers, or EHR-linked decision-support systems without increasing reporting time or creating unclear responsibility for model errors. Finally, cost-effectiveness studies are needed to determine whether radiomics reduces downstream imaging, prevents missed fractures, improves patient selection for intervention, or improves monitoring of metastatic and degenerative spine disease.

## 4 Conclusion

Radiomics holds significant potential for advancing the clinical assessment of the disease process and response to treatment, spinal pathology and for personalizing management of these conditions. Going beyond traditional diagnostic methods, radiomics provides a more detailed analysis of bone and intervertebral disc tissue structure and composition, enabling early detection of subtle fractures and degenerative changes that are not easily identified with conventional imaging techniques. This is particularly valuable for offering insights into bone microarchitecture related to osteoporosis and bone metastasis, evaluating tissue changes related to intervertebral disc herniation and low back pain, detecting undiagnosed osteoporotic vertebral fractures, common in aging populations, differentiating between acute and chronic fractures and predicting future fracture risks, all of which have eluded current clinical imaging analysis methods with a strong potential for enhancing clinical decision-making and supporting personalized treatment approaches. However, challenges remain, including the need for larger datasets, multicenter studies, and standardized imaging protocols to ensure the reproducibility and clinical utility of radiomic models. Addressing these limitations is crucial to fully integrating radiomics into routine practice, yet its potential to improve early detection and management of osteoporosis and spinal fractures offers promising advancements in patient care.

## References

[B1] Abdi H. Williams L. J. (2010). Principal component analysis. Wiley Interdiscip. Rev. Comput. Stat. 2 (4), 433–459. 10.1002/wics.101

[B2] Aerts H. J. Velazquez E. R. Leijenaar R. T. Parmar C. Grossmann P. Carvalho S. (2014). Decoding tumour phenotype by noninvasive imaging using a quantitative radiomics approach. Nat. Commun. 5, 4006. 10.1038/ncomms5006 24892406 PMC4059926

[B3] Alderson P. O. Summers R. M. (2020). The evolving status of radiomics. J. Natl. Cancer Inst. 112 (9), 869–870. 10.1093/jnci/djaa018 32016420 PMC7492766

[B4] Alzubaidi L. Zhang J. Humaidi A. J. Al-Dujaili A. Duan Y. Al-Shamma O. (2021). Review of deep learning: concepts, CNN architectures, challenges, applications, future directions. J. Big Data 8 (1), 53. 10.1186/s40537-021-00444-8 PMC8010506 33816053

[B5] Amelot A. Terrier L. M. Farah K. Aggad M. Le Nail L. R. Francois P. (2024). Impact of metastatic epidural spinal cord compression (MESCC) and pathological vertebral compression fracture (pVCF) in neurological and survival prognosis. Eur. J. Surg. Oncol. 50 (2), 107935. 10.1016/j.ejso.2023.107935 38199005

[B6] Avanzo M. Stancanello J. Pirrone G. Sartor G. (2020). Radiomics and deep learning in lung cancer. Strahlenther. Onkol. 196 (10), 879–887. 10.1007/s00066-020-01625-9 32367456

[B7] Badhwar A. Collin-Verreault Y. Orban P. Urchs S. Chouinard I. Vogel J. (2020). Multivariate consistency of resting-state fMRI connectivity maps acquired on a single individual over 2.5 years, 13 sites and 3 vendors. Neuroimage. 205, 116210. 10.1016/j.neuroimage.2019.116210 31593793

[B8] Bae Y. Kim M. Jeong W. Jang S. Lee S. W. (2025). Epidemiology and future burden of vertebral fractures: insights from the global burden of disease 1990-2021. Healthc. (Basel) 13 (15), Epub 20250722. 10.3390/healthcare13151774 PMC12346102 40805807

[B9] Bailey S. Hackney D. Vashishth D. Alkalay R. N. (2020). The effects of metastatic lesion on the structural determinants of bone: current clinical and experimental approaches. Bone 138, 115159. 10.1016/j.bone.2019.115159 31759204 PMC7531290

[B10] Bailey S. Stadelmann M. A. Zysset P. K. Vashishth D. Alkalay R. N. (2022). Influence of metastatic bone lesion type and tumor origin on human vertebral bone architecture, matrix quality, and mechanical properties. J. Bone Min. Res. 37 (5), 896–907. 10.1002/jbmr.4539 PMC9158727 35253282

[B11] Ballane G. Cauley J. A. Luckey M. M. El-Hajj F. G. (2017). Worldwide prevalence and incidence of osteoporotic vertebral fractures. Osteoporos. Int. 28 (5), 1531–1542. 10.1007/s00198-017-3909-3 28168409

[B12] Bell T. K. Godfrey K. J. Ware A. L. Yeates K. O. Harris A. D. (2022). Harmonization of multi-site MRS data with ComBat. Neuroimage. 257, 119330. 10.1016/j.neuroimage.2022.119330 35618196

[B13] Black D. M. Cummings S. R. Stone K. Hudes E. Palermo L. Steiger P. (1991). A new approach to defining normal vertebral dimensions. J. Bone Min. Res. 6 (8), 883–892. 10.1002/jbmr.5650060814 1785377

[B14] Blake G. M. Fogelman I. (2007). The role of DXA bone density scans in the diagnosis and treatment of osteoporosis. Postgrad. Med. J. 83 (982), 509–517. 10.1136/pgmj.2007.057505 17675543 PMC2600106

[B15] Bonaldi L. Pretto A. Pirri C. Uccheddu F. Fontanella C. G. Stecco C. (2023). Deep learning-based medical images segmentation of musculoskeletal anatomical structures: a survey of bottlenecks and strategies. Bioeng. (Basel) 10 (2), 137. 10.3390/bioengineering10020137 PMC9952222 36829631

[B16] Bradshaw T. J. Huemann Z. Hu J. Rahmim A. (2023). A guide to cross-validation for artificial intelligence in medical imaging. Radiol. Artif. Intell. 5 (4), e220232. 10.1148/ryai.220232 37529208 PMC10388213

[B17] Breiman L. (2001). Random forests. Random For. Mach. Learn. 45 (1), 5–32. 10.1023/a:1010933404324

[B18] Brooks F. J. Grigsby P. W. (2014). The effect of small tumor volumes on studies of intratumoral heterogeneity of tracer uptake. J. Nucl. Med. 55 (1), 37–42. 10.2967/jnumed.112.116715 24263086 PMC4017737

[B19] Busscher I. Ploegmakers J. J. Verkerke G. J. Veldhuizen A. G. (2010). Comparative anatomical dimensions of the complete human and porcine spine. Eur. Spine J. 19 (7), 1104–1114. 10.1007/s00586-010-1326-9 20186441 PMC2900026

[B20] Buvat I. Orlhac F. (2019). The dark side of radiomics: on the paramount importance of publishing negative results. J. Nucl. Med. 60 (11), 1543–1544. 10.2967/jnumed.119.235325 31541033

[B21] Cai J. Shen C. Yang T. Jiang Y. Ye H. Ruan Y. (2023). MRI-based radiomics assessment of the imminent new vertebral fracture after vertebral augmentation. Eur. Spine J. 32 (11), 3892–3905. 10.1007/s00586-023-07887-y 37624438

[B22] Cao J. Li Q. Zhang H. Wu Y. Wang X. Ding S. (2024). Radiomics model based on MRI to differentiate spinal multiple myeloma from metastases: a two-center study. J. Bone Oncol. 45, 100599. 10.1016/j.jbo.2024.100599 PMC11004638 38601920

[B23] Carrasquinha E. Santinha J. Mongolin A. Lisitskiya M. Ribeiro J. Cardoso F. (2019). “Regularization Techniques in Radiomics: A Case Study on the Prediction of Pcr in Breast Tumours and the Axilla.” in International Meeting on Computational Intelligence Methods for Bioinformatics and Biostatistics (Springer).

[B24] Carre A. Battistella E. Niyoteka S. Sun R. Deutsch E. Robert C. (2022). AutoComBat: a generic method for harmonizing MRI-based radiomic features. Sci. Rep. 12 (1), 12762. 10.1038/s41598-022-16609-1 35882891 PMC9325761

[B25] Chang Y. Lafata K. Sun W. Wang C. Chang Z. Kirkpatrick J. P. (2019). An investigation of machine learning methods in delta-radiomics feature analysis. PLoS One 14 (12), e0226348. 10.1371/journal.pone.0226348 31834910 PMC6910670

[B26] Chang C. Y. Buckless C. Yeh K. J. Torriani M. (2022). Automated detection and segmentation of sclerotic spinal lesions on body CTs using a deep convolutional neural network. Skelet. Radiol. 51 (2), 391–399. 10.1007/s00256-021-03873-x 34291325

[B27] Chattopadhay A. Sarkar A. Howlader P. Balasubramanian V. N. (2018). “Grad-cam++: generalized gradient-based visual explanations for deep convolutional networks,” 2018 IEEE Winter Conference on Applications of Computer Vision (WACV) (Cham, Switzerland: Springer).

[B28] Chee C. G. Yoon M. A. Kim K. W. Ko Y. Ham S. J. Cho Y. C. (2021). Combined radiomics-clinical model to predict malignancy of vertebral compression fractures on CT. Eur. Radiol. 31 (9), 6825–6834. 10.1007/s00330-021-07832-x 33742227

[B29] Chen T. Guestrin C. (2016). “Xgboost: a scalable tree boosting system,” Proceedings of the 22nd Acm Sigkdd International Conference on Knowledge Discovery and Data Mining. New York, NY: Association for Computing Machinery (ACM).

[B30] Chen K. Cao J. Zhang X. Wang X. Zhao X. Li Q. (2022). Differentiation between spinal multiple myeloma and metastases originated from lung using multi-view attention-guided network. Front. Oncol. 12, 981769. 10.3389/fonc.2022.981769 36158659 PMC9495278

[B31] Chen Y. Mo Y. Readie A. Ligozio G. Mandal I. Jabbar F. (2024). VertXNet: an ensemble method for vertebral body segmentation and identification from cervical and lumbar spinal X-rays. Sci. Rep. 14 (1), 3341. 10.1038/s41598-023-49923-3 38336974 PMC10858234

[B32] Chen B. Cui J. Li C. Xu P. Xu G. Jiang J. (2024). Application of radiomics model based on lumbar computed tomography in diagnosis of elderly osteoporosis. J. Orthop. Res. 42 (6), 1356–1368. 10.1002/jor.25789 38245854

[B33] Cheng L. Cai F. Xu M. Liu P. Liao J. Zong S. (2023). A diagnostic approach integrated multimodal radiomics with machine learning models based on lumbar spine CT and X-ray for osteoporosis. J. Bone Min. Metab. 41 (6), 877–889. 10.1007/s00774-023-01469-0 37898574

[B34] Cheng Q. Zhang J. Hu M. Wang S. Liu Y. Li J. (2024). Enhancing the opportunistic bone status assessment using radiomics based on dual-energy spectral CT material decomposition images. Bioeng. (Basel). 11 (12), 1257. 10.3390/bioengineering11121257 PMC11673124 39768075

[B35] Chianca V. Cuocolo R. Gitto S. Albano D. Merli I. Badalyan J. (2021). Radiomic machine learning classifiers in spine bone tumors: a multi-software, multi-scanner study. Eur. J. Radiol. 137, 109586. 10.1016/j.ejrad.2021.109586 33610852

[B36] Chiari-Correia N. S. Nogueira-Barbosa M. H. Chiari-Correia R. D. Azevedo-Marques P. M. (2023). A 3D Radiomics-Based artificial neural network model for benign *versus* malignant vertebral compression fracture classification in MRI. J. Digit. Imaging 36 (4), 1565–1577. 10.1007/s10278-023-00847-4 PMC10406770 37253895

[B37] Chiu C. K. Chin T. F. Chung W. H. Chan C. Y. W. Kwan M. K. (2024). Variations in the number of vertebrae, prevalence of Lumbosacral transitional vertebra and prevalence of cervical rib among surgical patients with adolescent idiopathic scoliosis: an analysis of 998 radiographs. Spine (Phila Pa 1976) 49 (1), 64–70. 10.1097/brs.0000000000004711 37146062

[B38] Coleman R. E. Croucher P. I. Padhani A. R. Clezardin P. Chow E. Fallon M. (2020). Bone metastases. Nat. Rev. Dis. Prim. 6 (1), 83. 10.1038/s41572-020-00216-3 33060614

[B39] Collins G. S. Reitsma J. B. Altman D. G. Moons K. G. (2015). Transparent reporting of a multivariable prediction model for individual prognosis or diagnosis (TRIPOD): the TRIPOD statement. Ann. Intern Med. 162 (1), 55–63. 10.7326/M14-0697 25560714

[B40] Confavreux C. B. Follet H. Mitton D. Pialat J. B. Clezardin P. (2021). Fracture risk evaluation of bone metastases: a burning issue. Cancers (Basel) 13 (22), 5711. 10.3390/cancers13225711 34830865 PMC8616502

[B41] Cortes C. Vapnik V. (1995). Support-vector networks. Mach. Learning 20 (3), 273–297. 10.1007/bf00994018

[B42] Cox D. R. (1972). Regression models and life-tables. J. R. Stat. Soc. Ser. B Methodol. 34 (2), 187–202. 10.1111/j.2517-6161.1972.tb00899.x

[B43] Cucchiara F. Petrini I. Romei C. Crucitta S. Lucchesi M. Valleggi S. (2021). Combining liquid biopsy and radiomics for personalized treatment of lung cancer patients. State of the art and new perspectives. Pharmacol. Res. 169, 105643. 10.1016/j.phrs.2021.105643 33940185

[B44] Cui L. Xu M. Liu C. Liu T. Yan X. Zhang Y. (2025). Towards reliable healthcare imaging: a multifaceted approach in class imbalance handling for medical image segmentation. Interdiscip. Sci. 17 (3), 614–633. 10.1007/s12539-025-00726-2 40624391 PMC12289783

[B45] Dai H. Wang Y. Fu R. Ye S. He X. Luo S. (2023). Radiomics and stacking regression model for measuring bone mineral density using abdominal computed tomography. Acta Radiol. 64 (1), 228–236. 10.1177/02841851211068149 34964365

[B46] Davis A. Gao R. Navin N. (2017). Tumor evolution: Linear, branching, neutral or punctuated? Biochim. Biophys. Acta Rev. Cancer 1867 (2), 151–161. 10.1016/j.bbcan.2017.01.003 28110020 PMC5558210

[B47] Del Lama R. S. Candido R. M. Chiari-Correia N. S. Nogueira-Barbosa M. H. de Azevedo-Marques P. M. Tinos R. (2022). Computer-aided diagnosis of vertebral compression fractures using convolutional neural networks and radiomics. J. Digit. Imaging 35 (3), 446–458. 10.1007/s10278-022-00586-y 35132524 PMC9156595

[B48] Delmas P. D. van de Langerijt L. Watts N. B. Eastell R. Genant H. Grauer A. (2005). Underdiagnosis of vertebral fractures is a worldwide problem: the IMPACT study. J. Bone Min. Res. 20 (4), 557–563. 10.1359/JBMR.041214 15765173

[B49] Demircioglu A. (2025). Reproducibility and interpretability in radiomics: a critical assessment. Diagn Interv. Radiol. 31 (4), 321–328. 10.4274/dir.2024.242719 PMC12239541 39463040

[B50] Diaz-Pinto A. Mehta P. Alle S. Asad M. Brown R. Nath V. (2022). “DeepEdit: deep editable learning for interactive segmentation of 3D medical images,” MICCAI Workshop on Data Augmentation, Labelling, and Imperfections (Springer).

[B51] Diaz-Pinto A. Alle S. Nath V. Tang Y. Ihsani A. Asad M. (2024). MONAI label: a framework for AI-assisted interactive labeling of 3D medical images. Med. Image Anal. 95, 103207. 10.1016/j.media.2024.103207 38776843

[B52] Do T. D. Sutter R. Skornitzke S. Weber M. A. (2018). CT and MRI techniques for imaging around orthopedic hardware. Rofo 190 (1), 31–41. 10.1055/s-0043-118127 28934809

[B53] Duan S. Hua Y. Cao G. Hu J. Cui W. Zhang D. (2023). Differential diagnosis of benign and malignant vertebral compression fractures: Comparison and correlation of radiomics and deep learning frameworks based on spinal CT and clinical characteristics. Eur. J. Radiol. 165, 110899. 10.1016/j.ejrad.2023.110899 37300935

[B54] Eertink J. J. Heymans M. W. Zwezerijnen G. J. C. Zijlstra J. M. de Vet H. C. W. Boellaard R. (2022). External validation: a simulation study to compare cross-validation *versus* holdout or external testing to assess the performance of clinical prediction models using PET data from DLBCL patients. EJNMMI Res. 12 (1), 58. 10.1186/s13550-022-00931-w 36089634 PMC9464671

[B55] Elliott D. M. Newman H. R. Conner M. N. Johnson C. L. Vresilovic E. J. (2026). Advances in mechanical assessments of *in vivo* human lumbar spine tissues with noninvasive imaging techniques. NPJ Biomed. Innov. 3 (1), 15. 10.1038/s44385-026-00070-0 41728629 PMC12916484

[B56] Faiella E. Santucci D. Calabrese A. Russo F. Vadala G. Zobel B. B. (2022). Artificial intelligence in bone metastases: an MRI and CT imaging review. Int. J. Environ. Res. Public Health 19 (3), 1880. 10.3390/ijerph19031880 35162902 PMC8834956

[B57] Fan Z. Wu T. Wang Y. Jin Z. Liu D. (2024). Deep-learning-based radiomics to predict surgical risk factors for lumbar disc herniation in young patients: a multicenter study. J. Multidiscip. Healthc. 17, 5991–6007. 10.2147/jmdh.s493302 39664265 PMC11633295

[B58] Fang K. Zheng X. Lin X. Dai Z. (2024). A comprehensive approach for osteoporosis detection through chest CT analysis and bone turnover markers: harnessing radiomics and deep learning techniques. Front. Endocrinol. (Lausanne) 15, 1296047. 10.3389/fendo.2024.1296047 PMC11183288 38894742

[B59] Fedorov A. Beichel R. Kalpathy-Cramer J. Finet J. Fillion-Robin J. C. Pujol S. (2012). 3D slicer as an image computing platform for the quantitative imaging network. Magn. Reson Imaging 30 (9), 1323–1341. 10.1016/j.mri.2012.05.001 22770690 PMC3466397

[B60] Feng Q. Xu S. Gong X. Wang T. He X. Liao D. (2024). An MRI-based radiomics nomogram for differentiation of benign and malignant vertebral compression fracture. Acad. Radiol. 31 (2), 605–616. 10.1016/j.acra.2023.07.011 37586940

[B61] Ferrar L. Jiang G. Adams J. Eastell R. (2005). Identification of vertebral fractures: an update. Osteoporos. Int. 16 (7), 717–728. 10.1007/s00198-005-1880-x 15868071

[B62] Fink H. A. Milavetz D. L. Palermo L. Nevitt M. C. Cauley J. A. Genant H. K. (2005). What proportion of incident radiographic vertebral deformities is clinically diagnosed and *vice versa?* J. Bone Min. Res. 20 (7), 1216–1222. 10.1359/JBMR.050314 15940375

[B63] Fix E. (1951). Discriminatory Analysis: Nonparametric Discrimination, Consistency Properties. Randolph Field, TX: USAF School of Aviation Medicine.

[B65] Frighetto-Pereira L. Rangayyan R. M. Metzner G. A. de Azevedo-Marques P. M. Nogueira-Barbosa M. H. (2016). Shape, texture and statistical features for classification of benign and malignant vertebral compression fractures in magnetic resonance images. Comput. Biol. Med. 73, 147–156. 10.1016/j.compbiomed.2016.04.006 27111110

[B66] Galbusera F. Cina A. O'Riordan D. Vitale J. A. Loibl M. Fekete T. F. (2024). Estimating lumbar bone mineral density from conventional MRI and radiographs with deep learning in spine patients. Eur. Spine J. 33 (11), 4092–4103. 10.1007/s00586-024-08463-8 39212711

[B67] Ge G. Zhang J. (2023). Feature selection methods and predictive models in CT lung cancer radiomics. J. Applied Clinical Medical Physics 24 (1), e13869. 10.1002/acm2.13869 36527376 PMC9860004

[B68] Gehlbach S. H. Bigelow C. Heimisdottir M. May S. Walker M. Kirkwood J. R. (2000). Recognition of vertebral fracture in a clinical setting. Osteoporos. Int. 11 (7), 577–582. 10.1007/s001980070078 11069191

[B69] Genant H. K. Wu C. Y. van Kuijk C. Nevitt M. C. (1993). Vertebral fracture assessment using a semiquantitative technique. J. Bone Min. Res. 8 (9), 1137–1148. 10.1002/jbmr.5650080915 8237484

[B70] Genant H. K. Cooper C. Poor G. Reid I. Ehrlich G. Kanis J. (1999). Interim report and recommendations of the World Health Organization task-force for osteoporosis. Osteoporos. Int. 10 (4), 259–264. 10.1007/s001980050224 10692972

[B71] Geng W. Zhu J. Li M. Pi B. Wang X. Xing J. (2024). Radiomics based on multimodal magnetic resonance imaging for the differential diagnosis of benign and malignant vertebral compression fractures. Orthop. Surg. 16 (10), 2464–2474. 10.1111/os.14148 38982652 PMC11456728

[B72] Gillies R. J. Kinahan P. E. Hricak H. (2016). Radiomics: images are more than pictures, they are data. Radiology 278 (2), 563–577. 10.1148/radiol.2015151169 26579733 PMC4734157

[B73] Gitto S. Bologna M. Corino V. D. A. Emili I. Albano D. Messina C. (2022). Diffusion-weighted MRI radiomics of spine bone tumors: feature stability and machine learning-based classification performance. Radiol. Med. 127 (5), 518–525. 10.1007/s11547-022-01468-7 35320464 PMC9098537

[B74] Grigoryan M. Guermazi A. Roemer F. W. Delmas P. D. Genant H. K. (2003). Recognizing and reporting osteoporotic vertebral fractures. Eur. Spine J. 12 (2), S104–S112. 10.1007/s00586-003-0613-0 13680316 PMC3591834

[B75] Gui C. Chen X. Sheikh K. Mathews L. Lo S. L. Lee J. (2022). Radiomic modeling to predict risk of vertebral compression fracture after stereotactic body radiation therapy for spinal metastases. J. Neurosurg. Spine 36 (2), 294–302. 10.3171/2021.3.spine201534 34560656

[B76] Haarburger C. Müller-Franzes G. Weninger L. Kuhl C. Truhn D. Merhof D. (2020). Radiomics feature reproducibility under inter-rater variability in segmentations of CT images. Sci. Reports 10 (1), 12688. 10.1038/s41598-020-69534-6 32728098 PMC7391354

[B77] Haouchine N. Hackney D. B. Pieper S. D. Wells I. W. M. Sanhinova M. Balboni T. A. (2024). An open annotated dataset and baseline machine learning model for segmentation of vertebrae with metastatic bone lesions from CT. medRxiv.

[B78] Hastie T. (2009). The Elements of Statistical Learning: Data Mining, Inference, and Prediction. Springer.

[B79] Hastie T. Tibshirani R. Friedman J. H. Friedman J. H. (2009). The Elements of Statistical Learning: Data Mining, Inference, and Prediction. Springer.

[B80] He L. Liu Z. Liu C. Gao Z. Ren Q. Lei L. (2021). Radiomics based on lumbar spine magnetic resonance imaging to detect osteoporosis. Acad. Radiol. 28 (6), e165–e171. 10.1016/j.acra.2020.03.046 32386949

[B81] Heckel F. Moltz J. H. Meine H. Geisler B. Kiessling A. D'Anastasi M. (2014). On the evaluation of segmentation editing tools. J. Med. Imaging (Bellingham) 1 (3), 034005. 10.1117/1.JMI.1.3.034005 26158063 PMC4478728

[B82] Hille G. Saalfeld S. Serowy S. Tonnies K. (2018). Vertebral body segmentation in wide range clinical routine spine MRI data. Comput. Methods Programs Biomed. 155, 93–99. 10.1016/j.cmpb.2017.12.013 29512508

[B83] Hinzpeter R. Baumann L. Guggenberger R. Huellner M. Alkadhi H. Baessler B. (2022). Radiomics for detecting prostate cancer bone metastases invisible in CT: a proof-of-concept study. Eur. Radiol. 32 (3), 1823–1832. 10.1007/s00330-021-08245-6 PMC8831270 34559264

[B84] Ho T. K. (1998). The random subspace method for constructing decision forests. IEEE Transactions Pattern Analysis Machine Intelligence 20 (8), 832–844. 10.1109/34.709601

[B85] Hoerl A. E. Kennard R. W. (1970). Ridge regression: biased estimation for nonorthogonal problems. Technometrics 12 (1), 55–67. 10.1080/00401706.1970.10488634

[B86] Hong L. Zhou Z. Xu W. Wang P. Wei B. (2026). Graph-guided frequency-enhanced state space network for 3D spine segmentation from MR images. J. Appl. Clin. Med. Phys. 27 (2), e70481. 10.1002/acm2.70481 41679913 PMC12900570

[B87] Hooten W. M. Cohen S. P. (2015). Evaluation and treatment of low back pain: a clinically focused review for primary care specialists. Mayo Clin. Proc. 90 (12), 1699–1718. 10.1016/j.mayocp.2015.10.009 26653300

[B88] Horng H. Singh A. Yousefi B. Cohen E. A. Haghighi B. Katz S. (2022). Generalized ComBat harmonization methods for radiomic features with multi-modal distributions and multiple batch effects. Sci. Rep. 12 (1), 4493. 10.1038/s41598-022-08412-9 35296726 PMC8927332

[B89] Hosny A. Aerts H. J. Mak R. H. (2019). Handcrafted *versus* deep learning radiomics for prediction of cancer therapy response. Lancet Digital Health 1 (3), e106–e107. 10.1016/S2589-7500(19)30062-7 33323257

[B90] Hoy D. March L. Brooks P. Blyth F. Woolf A. Bain C. (2014). The global burden of low back pain: estimates from the Global Burden of Disease 2010 study. Ann. Rheum. Dis. 73 (6), 968–974. 10.1136/annrheumdis-2013-204428 24665116

[B91] Hsieh H. C. Yen H. K. Tseng T. E. Pan Y. T. Liao M. T. Fu S. H. (2023). Determining patients with spinal metastases suitable for surgical intervention: a cost-effective analysis. Cancer Med. 12 (19), 20059–20069. 10.1002/cam4.6576 37749979 PMC10587930

[B92] Huo T. Wu W. Chen X. Xue M. Liu P. Zhang J. (2025). Deep learning-based multimodal data fusion in bone tumor management: advances in clinical decision support. Intell. Oncol. 1 (3), 204–215. 10.1016/j.intonc.2025.06.005

[B93] Isensee F. Jaeger P. F. Kohl S. A. A. Petersen J. Maier-Hein K. H. (2021). nnU-Net: a self-configuring method for deep learning-based biomedical image segmentation. Nat. Methods 18 (2), 203–211. 10.1038/s41592-020-01008-z 33288961

[B94] James G. Witten D. Hastie T. Tibshirani R. (2013). An Introduction to Statistical Learning: With Applications in R. Springer.

[B95] Jha A. K. Sherkhane U. B. Mthun S. Jaiswar V. Purandare N. Prabhash K. (2023). External validation of robust radiomic signature to predict 2-Year overall survival in non-small-cell lung cancer. J. Digit. Imaging 36 (6), 2519–2531. 10.1007/s10278-023-00835-8 37735307 PMC10584779

[B96] Jiang L. Liu X. G. Yuan H. S. Yang S. M. Li J. Wei F. (2014). Diagnosis and treatment of vertebral hemangiomas with neurologic deficit: a report of 29 cases and literature review. Spine J. 14 (6), 944–954. 10.1016/j.spinee.2013.07.450 24119881

[B97] Jiang Y. W. Xu X. J. Wang R. Chen C. M. (2022). Radiomics analysis based on lumbar spine CT to detect osteoporosis. Eur. Radiol. 32 (11), 8019–8026. 10.1007/s00330-022-08805-4 35499565 PMC9059457

[B98] Jiang Y. Cai J. Zeng Y. Ye H. Yang T. Liu Z. (2023a). Development and validation of a machine learning model to predict imminent new vertebral fractures after vertebral augmentation. BMC Musculoskelet. Disord. 24 (1), 472. 10.1186/s12891-023-06557-w 37296426 PMC10251538

[B99] Jiang Y. Zhang W. Huang S. Huang Q. Ye H. Zeng Y. (2023b). Preoperative prediction of new vertebral fractures after vertebral augmentation with a radiomics nomogram. Diagn. (Basel) 13 (22), 20231116. 10.3390/diagnostics13223459 PMC10670105 37998595

[B100] Jiménez E. G. Ferre J. Á. Cano M. P. Jiménez M. N. Cordero M. A. Fernández J. T. (2009). Osteoporosis involutiva tipo I en la mujer posmenopáusica: diagnóstico y manejo clínico. Rev. Española Enfermedades Metabólicas Óseas 18 (4), 77–84. 10.1016/S1132-8460(09)73462-2

[B101] Jin Z. Wang Y. Wang Y. Mao Y. Zhang F. Yu J. (2022). Application of 18F-FDG PET-CT images based radiomics in identifying vertebral multiple myeloma and bone metastases. Front. Med. (Lausanne) 9, 874847. 10.3389/fmed.2022.874847 35510246 PMC9058063

[B102] Jolliffe I. T. (2006). Principal Component Analysis. New York, NY: Springer New York.

[B103] Jovicich J. Minati L. Marizzoni M. Marchitelli R. Sala-Llonch R. Bartres-Faz D. (2016). Longitudinal reproducibility of default-mode network connectivity in healthy elderly participants: a multicentric resting-state fMRI study. Neuroimage 124 (Pt A), 442–454. 10.1016/j.neuroimage.2015.07.010 26163799

[B104] Kado D. M. Browner W. S. Palermo L. Nevitt M. C. Genant H. K. Cummings S. R. (1999). Vertebral fractures and mortality in older women: a prospective study. Archives Internal Medicine 159 (11), 1215–1220. 10.1001/archinte.159.11.1215 10371229

[B105] Kang S. R. Wang K. (2024). Radiomic nomogram based on lumbar spine magnetic resonance images to diagnose osteoporosis. Acta Radiol. 65 (8), 950–958. 10.1177/02841851241242052 38651258

[B106] Keaveny T. M. Morgan E. F. Niebur G. L. Yeh O. C. (2001). Biomechanics of trabecular bone. Annu. Rev. Biomed. Eng. 3, 307–333. 10.1146/annurev.bioeng.3.1.307 11447066

[B107] Khosla S. Lufkin E. G. Hodgson S. F. Fitzpatrick L. A. Melton L. J. 3rd (1994). Epidemiology and clinical features of osteoporosis in young individuals. Bone 15 (5), 551–555. 10.1016/8756-3282(94)90280-1 7980966

[B108] Kim A. Y. Yoon M. A. Ham S. J. Cho Y. C. Ko Y. Park B. (2022). Prediction of the acuity of vertebral compression fractures on CT using radiologic and radiomic features. Acad. Radiol. 29 (10), 1512–1520. 10.1016/j.acra.2021.12.008 34998683

[B109] Kleene S. C. (1951). Representation of Events in Nerve Nets and Finite Automata. Santa Monica, CA: RAND Corporation.

[B110] Kocak B. Baessler B. Bakas S. Cuocolo R. Fedorov A. Maier-Hein L. (2023). CheckList for EvaluAtion of Radiomics research (CLEAR): a step-by-step reporting guideline for authors and reviewers endorsed by ESR and EuSoMII. Insights Imaging 14 (1), 75. 10.1186/s13244-023-01415-8 37142815 PMC10160267

[B111] Kohavi R. (1995). A Study of cross-validation and Bootstrap for Accuracy Estimation and Model Selection. Montreal, Canada: IJCAI.

[B112] Korez R. Ibragimov B. Likar B. Pernus F. Vrtovec T. (2015). A framework for automated spine and vertebrae interpolation-based detection and model-based segmentation. IEEE Trans. Med. Imaging 34 (8), 1649–1662. 10.1109/TMI.2015.2389334 25585415

[B113] Kubota T. Yamada K. Ito H. Kizu O. Nishimura T. (2005). High-resolution imaging of the spine using multidetector-row computed tomography: differentiation between benign and malignant vertebral compression fractures. J. Comput. Assist. Tomogr. 29 (5), 712–719. 10.1097/01.rct.0000175500.41836.24 16163049

[B114] Lambin P. Rios-Velazquez E. Leijenaar R. Carvalho S. van Stiphout R. G. Granton P. (2012). Radiomics: extracting more information from medical images using advanced feature analysis. Eur. J. Cancer 48 (4), 441–446. 10.1016/j.ejca.2011.11.036 22257792 PMC4533986

[B115] Lewiecki E. M. Laster A. J. (2006). Clinical review: clinical applications of vertebral fracture assessment by dual-energy x-ray absorptiometry. J. Clin. Endocrinol. Metab. 91 (11), 4215–4222. 10.1210/jc.2006-1178 16940447

[B116] Li Z. Guan M. Sun D. Xu Y. Li F. Xiong W. (2018). A novel MRI- and CT-based scoring system to differentiate malignant from osteoporotic vertebral fractures in Chinese patients. BMC Musculoskelet. Disord. 19 (1), 406. 10.1186/s12891-018-2331-0 30458738 PMC6247741

[B117] Li K. Huang L. Lang Z. Ni L. Du J. Yang H. (2019). Reliability and validity of different MRI sequences in improving the accuracy of differential diagnosis of benign and malignant vertebral fractures: a meta-analysis. Am. J. Roentgenol. 213 (2), 427–436. 10.2214/AJR.18.20560 31039028

[B118] Li W. G. Zeng R. Lu Y. Li W. X. Wang T. T. Lin H. (2023). The value of radiomics-based CT combined with machine learning in the diagnosis of occult vertebral fractures. BMC Musculoskelet. Disord. 24 (1), 819. 10.1186/s12891-023-06939-0 37848859 PMC10580519

[B119] Li S. Yu X. Shi R. Zhu B. Zhang R. Kang B. (2023). MRI-based radiomics nomogram for differentiation of solitary metastasis and solitary primary tumor in the spine. BMC Med. Imaging 23 (1), 29. 10.1186/s12880-023-00978-8 36755233 PMC9909949

[B120] Lin P. Yang P. F. Chen S. Shao Y. Y. Xu L. Wu Y. (2020). A Delta-radiomics model for preoperative evaluation of neoadjuvant chemotherapy response in high-grade osteosarcoma. Cancer Imaging 20 (1), 7. 10.1186/s40644-019-0283-8 31937372 PMC6958668

[B121] Lin X. Shen R. Zheng X. Shi S. Dai Z. Fang K. (2024). Utilizing radiomics techniques to isolate a single vertebral body from chest CT for opportunistic osteoporosis screening. BMC Musculoskelet. Disord. 25 (1), 785. 10.1186/s12891-024-07903-2 39367356 PMC11451174

[B122] Lin A. Zhang H. Wang Y. Cui Q. Zhu K. Zhou D. (2024). Radiomics based on MRI to predict recurrent L4-5 disc herniation after percutaneous endoscopic lumbar discectomy. BMC Med. Imaging 24 (1), 273. 10.1186/s12880-024-01450-x 39390384 PMC11468133

[B123] Liu Z. Jiang Z. Meng L. Yang J. Liu Y. Zhang Y. (2021). Handcrafted and deep learning-based radiomic models can distinguish GBM from brain metastasis. J. Oncol. 2021, 5518717-10. 10.1155/2021/5518717 PMC8195660 34188680

[B124] Liu J. Guo W. Zeng P. Geng Y. Liu Y. Ouyang H. (2022). Vertebral MRI-based radiomics model to differentiate multiple myeloma from metastases: influence of features number on logistic regression model performance. Eur. Radiol. 32 (1), 572–581. 10.1007/s00330-021-08150-y 34255157

[B125] Liu J. Tang J. Xia B. Gu Z. Yin H. Zhang H. (2023). Novel Radiomics-Clinical model for the noninvasive prediction of new fractures after vertebral augmentation. Acad. Radiol. 30 (6), 1092–1100. 10.1016/j.acra.2022.06.022 35915030

[B126] Liu J. Wang H. Shan X. Zhang L. Cui S. Shi Z. (2024). Hybrid transformer convolutional neural network-based radiomics models for osteoporosis screening in routine CT. BMC Med. Imaging 24 (1), 62. 10.1186/s12880-024-01240-5 PMC10938662 38486185

[B127] Lodwick G. S. Wilson A. J. Farrell C. Virtama P. Dittrich F. (1980). Determining growth rates of focal lesions of bone from radiographs. Radiology 134 (3), 577–583. 10.1148/radiology.134.3.6928321 6928321

[B128] Lou B. Doken S. Zhuang T. Wingerter D. Gidwani M. Mistry N. (2019). An image-based deep learning framework for individualising radiotherapy dose: a retrospective analysis of outcome prediction. Lancet Digital Health 1 (3), e136–e147. 10.1016/s2589-7500(19)30058-5 31448366 PMC6708276

[B129] Lu H. Arshad M. Thornton A. Avesani G. Cunnea P. Curry E. (2019). A mathematical-descriptor of tumor-mesoscopic-structure from computed-tomography images annotates prognostic- and molecular-phenotypes of epithelial ovarian cancer. Nat. Commun. 10 (1), 764. 10.1038/s41467-019-08718-9 30770825 PMC6377605

[B130] Mackin D. Fave X. Zhang L. Yang J. Jones A. K. Ng C. S. (2017). Harmonizing the pixel size in retrospective computed tomography radiomics studies. PLoS One 12 (9), e0178524. 10.1371/journal.pone.0178524 28934225 PMC5608195

[B131] Madzia-Madzou D. K. Jak M. de Keizer B. Verlaan J. J. Minnema M. C. Gilhuijs K. (2025). Automated vertebrae identification and segmentation with structural uncertainty analysis in longitudinal CT scans of patients with multiple myeloma. Eur. J. Radiol. 188, 112160. 10.1016/j.ejrad.2025.112160 40349413

[B132] Mahon R. N. Ghita M. Hugo G. D. Weiss E. (2020). ComBat harmonization for radiomic features in independent phantom and lung cancer patient computed tomography datasets. Phys. Med. Biol. 65 (1), 015010. 10.1088/1361-6560/ab6177 31835261

[B133] Mannil M. von Spiczak J. Manka R. Alkadhi H. (2018). Texture analysis and machine learning for detecting myocardial infarction in noncontrast low-dose computed tomography: unveiling the invisible. Invest Radiol. 53 (6), 338–343. 10.1097/RLI.0000000000000448 29420321

[B134] Masokano I. B. Liu W. Xie S. Marcellin D. F. H. Pei Y. Li W. (2020). The application of texture quantification in hepatocellular carcinoma using CT and MRI: a review of perspectives and challenges. Cancer Imaging 20 (1), 67. 10.1186/s40644-020-00341-y 32962762 PMC7510095

[B135] Matos R. Fernandes P. R. Matela N. Castro A. P. G. (2023). Lumbar intervertebral disc segmentation for computer modeling and simulation. Comput. Methods Programs Biomed. 230, 107337. 10.1016/j.cmpb.2023.107337 36634387

[B136] Mauch J. T. Carr C. M. Cloft H. Diehn F. E. (2018). Review of the imaging features of benign osteoporotic and malignant vertebral compression fractures. Am. J. Neuroradiol. 39 (9), 1584–1592. 10.3174/ajnr.A5528 29348133 PMC7655272

[B137] McCullagh K. Zamora C. Castillo M. (2023). Troublemaking lesions: spinal tumor mimics. Neuroimaging Clin. N. Am. 33 (3), 423–441. 10.1016/j.nic.2023.03.003 37356860

[B138] McLachlan G. J. (2005). Discriminant Analysis and Statistical Pattern Recognition. John Wiley & Sons.

[B139] McSweeney T. Tiulpin A. Kowlagi N. Määttä J. Karppinen J. Saarakkala S. (2025). Robust radiomic signatures of intervertebral disc degeneration from MRI. Spine 50 (24), 1737–1746. 10.1097/brs.0000000000005435 40539398 PMC12637164

[B140] Mesguich C. Hindie E. de Senneville B. D. Tlili G. Pinaquy J. B. Marit G. (2021). Improved 18-FDG PET/CT diagnosis of multiple myeloma diffuse disease by radiomics analysis. Nucl. Med. Commun. 42 (10), 1135–1143. 10.1097/MNM.0000000000001437 34001823

[B141] Miles K. (2020). Radiomics for personalised medicine: the long road ahead. Br. J. Cancer 122 (7), 929–930. 10.1038/s41416-019-0699-8 31937924 PMC7109132

[B142] Misir A. (2025). Artificial intelligence in orthopedic trauma: a comprehensive review. Injury 56 (8), 112570. 10.1016/j.injury.2025.112570 40683054

[B143] Mohammadi-Sadr M. Cheki M. Moslehi M. Zarasvandnia M. Salamat M. R. (2025). A novel approach based on integrating radiomics, bone morphometry and Hounsfield unit-derived from routine chest CT for bone mineral density assessment. Acad. Radiol. 32 (4), 2284–2296. 10.1016/j.acra.2024.10.049 39562197

[B144] Montoya C. Spieler B. Welford S. M. Kwon D. Pra A. D. Lopes G. (2023). Predicting response to immunotherapy in non-small cell lung cancer-from bench to bedside. Front. Oncol. 13, 1225720. 10.3389/fonc.2023.1225720 38033493 PMC10686412

[B145] Morimoto T. Toda Y. Hakozaki M. Paholpak P. Watanabe K. Kato K. (2024). A new era in the management of spinal metastasis. Front. Oncol. 14, 1374915. 10.3389/fonc.2024.1374915 38694784 PMC11062132

[B146] Nardone V. Reginelli A. Grassi R. Boldrini L. Vacca G. D'Ippolito E. (2021). Delta radiomics: a systematic review. Radiol. Med. 126 (12), 1571–1583. 10.1007/s11547-021-01436-7 34865190

[B147] Naseri H. Skamene S. Tolba M. Faye M. D. Ramia P. Khriguian J. (2022). Radiomics-based machine learning models to distinguish between metastatic and healthy bone using lesion-center-based geometric regions of interest. Sci. Rep. 12 (1), 9866. 10.1038/s41598-022-13379-8 35701461 PMC9198102

[B148] Naseri H. Skamene S. Tolba M. Faye M. D. Ramia P. Khriguian J. (2023). A scalable Radiomics- and natural language processing-based machine learning pipeline to distinguish between painful and painless thoracic spinal bone metastases: retrospective algorithm development and validation study. JMIR AI 2, e44779. 10.2196/44779 38875572 PMC11041487

[B149] Nelder J. A. Wedderburn R. W. (1972). Generalized linear models. J. R. Stat. Soc. Ser. A Statistics Soc. 135 (3), 370–384. 10.2307/2344614

[B150] Ngo K. Patil P. McGowan S. J. Niedernhofer L. J. Robbins P. D. Kang J. (2017). Senescent intervertebral disc cells exhibit perturbed matrix homeostasis phenotype. Mech. Ageing Dev. 166, 16–23. 10.1016/j.mad.2017.08.007 28830687 PMC5632945

[B151] Nian S. Zhao Y. Li C. Zhu K. Li N. Li W. (2024). Development and validation of a radiomics-based model for predicting osteoporosis in patients with lumbar compression fractures. Spine J. 24 (9), 1625–1634. 10.1016/j.spinee.2024.04.016 38679078

[B152] Nwogu I. Corso J. J. (2008). Exploratory identification of image-based biomarkers for solid mass pulmonary tumors. Med. Image Comput. Comput. Assist. Interv. 11 (1), 612–619. 10.1007/978-3-540-85988-8_73 18979797

[B153] Orlhac F. Eertink J. J. Cottereau A. S. Zijlstra J. M. Thieblemont C. Meignan M. (2022). A guide to ComBat harmonization of imaging biomarkers in multicenter studies. J. Nucl. Med. 63 (2), 172–179. 10.2967/jnumed.121.262464 34531263 PMC8805779

[B154] Oster G. Lamerato L. Glass A. G. Richert-Boe K. E. Lopez A. Chung K. (2013). Natural history of skeletal-related events in patients with breast, lung, or prostate cancer and metastases to bone: a 15-year study in two large US health systems. Support Care Cancer 21 (12), 3279–3286. 10.1007/s00520-013-1887-3 23884473

[B155] Pan J. Lin P. C. Gong S. C. Wang Z. Cao R. Lv Y. (2024). Feasibility study of opportunistic osteoporosis screening on chest CT using a multi-feature fusion DCNN model. Arch. Osteoporos. 19 (1), 98. 10.1007/s11657-024-01455-7 39414670 PMC11485148

[B156] Pan Y. Wan Y. Wu Y. Lin C. Ye Q. Liu J. (2024). Radiomics models based on thoracic and upper lumbar spine in chest LDCT to predict low bone mineral density. Sci. Rep. 14 (1), 31323. 10.1038/s41598-024-82642-x 39732811 PMC11682441

[B157] Papanikolaou N. Matos C. Koh D. M. (2020). How to develop a meaningful radiomic signature for clinical use in oncologic patients. Cancer Imaging 20 (1), 33. 10.1186/s40644-020-00311-4 32357923 PMC7195800

[B158] Park T. Yoon M. A. Cho Y. C. Ham S. J. Ko Y. Kim S. (2022). Automated segmentation of the fractured vertebrae on CT and its applicability in a radiomics model to predict fracture malignancy. Sci. Rep. 12 (1), 6735. 10.1038/s41598-022-10807-7 35468985 PMC9038736

[B159] Parmar C. Grossmann P. Bussink J. Lambin P. Aerts H. (2015). Machine learning methods for quantitative radiomic biomarkers. Sci. Rep. 5, 13087. 10.1038/srep13087 26278466 PMC4538374

[B160] Patel K. Cooper P. Belani P. Doshi A. (2025). Artificial intelligence in spine imaging: a paradigm shift in diagnosis and care. Magn. Reson Imaging Clin. N. Am. 33 (2), 389–398. 10.1016/j.mric.2025.01.001 40287253

[B161] Patil P. Dong Q. Wang D. Chang J. Wiley C. Demaria M. (2019). Systemic clearance of p16(INK4a) -positive senescent cells mitigates age-associated intervertebral disc degeneration. Aging Cell 18 (3), e12927. 10.1111/acel.12927 30900385 PMC6516165

[B162] Paul C. P. L. Smit T. H. de Graaf M. Holewijn R. M. Bisschop A. van de Ven P. M. (2018). Quantitative MRI in early intervertebral disc degeneration: T1rho correlates better than T2 and ADC with biomechanics, histology and matrix content. PLoS One 13 (1), e0191442. 10.1371/journal.pone.0191442 29381716 PMC5790235

[B163] Pfirrmann C. W. Metzdorf A. Zanetti M. Hodler J. Boos N. (2001). Magnetic resonance classification of lumbar intervertebral disc degeneration. Spine 26 (17), 1873–1878. 10.1097/00007632-200109010-00011 11568697

[B164] Poldrack R. A. Gorgolewski K. J. (2014). Making big data open: data sharing in neuroimaging. Nat. Neurosci. 17 (11), 1510–1517. 10.1038/nn.3818 25349916

[B165] Qin Z. Yu F. Liu C. Chen X. (2018). How convolutional neural network see the world-A survey of convolutional neural network visualization methods. arXiv preprint arXiv:180411191.

[B166] Qin H. Wu Y. Q. Lin P. Gao R. Z. Li X. Wang X. R. (2021). Ultrasound image-based radiomics: an innovative method to identify primary tumorous sources of liver metastases. J. Ultrasound Med. 40 (6), 1229–1244. 10.1002/jum.15506 32951217

[B167] Quinlan J. R. (1987). Simplifying decision trees. Int. Journal Man-Machine Studies 27 (3), 221–234. 10.1016/s0020-7373(87)80053-6

[B168] Quintiens J. Paravisi E. Uniyal P. van Lenthe G. H. (2025). Bone stiffness and strength at the distal radius can be determined using photon-counting CT. Arch. Osteoporos. 20 (1), 40. 10.1007/s11657-025-01527-2 40116966

[B169] Quintiens J. Morren T. Coudyzer W. van Lenthe G. H. (2026). Anisotropic mechanical characterization of trabecular bone with linear and non-linear voxel-based finite elements from photon-counting CT. Bone 202, 117683. 10.1016/j.bone.2025.117683 41092994

[B170] Ramlee S. Manavaki R. Aloj L. Escudero Sanchez L. (2024). Mitigating the impact of image processing variations on tumour [(18)F]-FDG-PET radiomic feature robustness. Sci. Rep. 14 (1), 16294. 10.1038/s41598-024-67239-8 39009706 PMC11251269

[B171] Reuze S. Schernberg A. Orlhac F. Sun R. Chargari C. Dercle L. (2018). Radiomics in nuclear medicine applied to radiation therapy: methods, pitfalls, and challenges. Int. J. Radiat. Oncol. Biol. Phys. 102 (4), 1117–1142. 10.1016/j.ijrobp.2018.05.022 30064704

[B172] Reznikov N. Buss D. J. Provencher B. McKee M. D. Piche N. (2020). Deep learning for 3D imaging and image analysis in biomineralization research. J. Struct. Biol. 212 (1), 107598. 10.1016/j.jsb.2020.107598 32783967

[B173] Rich J. M. Bhardwaj L. N. Shah A. Gangal K. Rapaka M. S. Oberai A. A. (2023). Deep learning image segmentation approaches for malignant bone lesions: a systematic review and meta-analysis. Front. Radiol. 3, 1241651. 10.3389/fradi.2023.1241651 37614529 PMC10442705

[B174] Riley R. D. Ensor J. Snell K. I. Archer L. Whittle R. Dhiman P. (2025). Importance of sample size on the quality and utility of AI-based prediction models for healthcare. Lancet Digital Health 7 (6), 100857. 10.1016/j.landig.2025.01.013 40461350

[B175] Rish I. (2001). “An empirical study of the naive Bayes classifier,” IJCAI 2001 Workshop on Empirical Methods in Artificial Intelligence (Seattle, USA).

[B176] Rizzo S. Botta F. Raimondi S. Origgi D. Fanciullo C. Morganti A. G. (2018). Radiomics: the facts and the challenges of image analysis. Eur. Radiol. Exp. 2 (1), 36. 10.1186/s41747-018-0068-z 30426318 PMC6234198

[B177] Ronneberger O. Fischer P. Brox T. (2015). “U-net: convolutional networks for biomedical image segmentation,” International Conference on Medical Image Computing and computer-assisted Intervention (Springer).

[B178] Russell S. J. Norvig P. (2021). Artificial Intelligence: A Modern Approach. Global Edition 4e. Harlow, United Kingdom: Pearson Education Limited.

[B179] Saad F. Lipton A. Cook R. Chen Y. M. Smith M. Coleman R. (2007). Pathologic fractures correlate with reduced survival in patients with malignant bone disease. Cancer 110 (8), 1860–1867. 10.1002/cncr.22991 17763372

[B180] Saeed M. U. Dikaios N. Dastgir A. Ali G. Hamid M. Hajjej F. (2023). An automated deep learning approach for spine segmentation and vertebrae recognition using computed tomography images. Diagn. (Basel) 13 (16), 2658. 10.3390/diagnostics13162658 PMC10453471 37627917

[B181] Sambri A. Fiore M. Giannini C. Pipola V. Zucchini R. Aparisi Gomez M. P. (2022). Primary tumors of the sacrum: imaging findings. Curr. Med. Imaging 18 (2), 170–186. 10.2174/1573405617666210512011923 33982654

[B182] Sanker V. Gowda P. Thaller A. Li Z. Heesen P. Qiang Z. (2025). Applications and performance of artificial intelligence in spinal metastasis imaging: a systematic review. J. Clin. Med. 14 (16), 5877. 10.3390/jcm14165877 40869703 PMC12387603

[B183] Sanli I. Osong B. Dekker A. TerHaag K. van Kuijk S. M. J. van Soest J. (2022). Radiomics biopsy signature for predicting survival in patients with spinal bone metastases (SBMs). Clin. Transl. Radiat. Oncol. 33, 57–65. 10.1016/j.ctro.2021.12.011 35079642 PMC8777154

[B184] Saravi B. Zink A. Ülkümen S. Couillard-Despres S. Wollborn J. Lang G. (2023). Clinical and radiomics feature-based outcome analysis in lumbar disc herniation surgery. BMC Musculoskelet. Disord. 24 (1), 791. 10.1186/s12891-023-06911-y 37803313 PMC10557221

[B185] Saravi B. Zink A. Tabukashvili E. Guzel H. E. Ulkumen S. Couillard-Despres S. (2024). Integrating radiomics with clinical data for enhanced prediction of vertebral fracture risk. Front. Bioeng. Biotechnol. 12, 1485364. 10.3389/fbioe.2024.1485364 39650236 PMC11620855

[B186] Sasaki T. Hata Y. Ando Y. Ishikawa M. Ishikawa H. (1999). “Fuzzy rule-based approach to segment the menisci regions from MR images,” Medical Imaging 1999: Image Processing (Bellingham, WA: SPIE).

[B187] Schroeder G. D. Guyre C. A. Vaccaro A. R. (2016). “The epidemiology and pathophysiology of lumbar disc herniations,” Seminars in Spine Surgery (Elsevier).

[B188] Schwaiger B. J. Gersing A. S. Baum T. Krestan C. R. Kirschke J. S. (2016). Distinguishing benign and malignant vertebral fractures using CT and MRI. Semin. Musculoskelet. Radiol. 20 (4), 345–352. 10.1055/s-0036-1592433 27842427

[B189] Sciubba D. M. Petteys R. J. Dekutoski M. B. Fisher C. G. Fehlings M. G. Ondra S. L. (2010). Diagnosis and management of metastatic spine disease. A review. J. Neurosurg. Spine 13 (1), 94–108. 10.3171/2010.3.SPINE09202 20594024

[B190] Seol Y. Song J. H. Choi K. H. Lee Y. K. Choi B. O. Kang Y. N. (2024). Predicting vertebral compression fracture prior to spinal SBRT using radiomics from planning CT. Eur. Spine J. 33 (8), 3221–3229. 10.1007/s00586-023-07963-3 37814013

[B191] Shi L. Rong Y. Daly M. Dyer B. Benedict S. Qiu J. (2020). Cone-beam computed tomography-based delta-radiomics for early response assessment in radiotherapy for locally advanced lung cancer. Phys. Med. Biol. 65 (1), 015009. 10.1088/1361-6560/ab3247 31307024

[B192] Shinohara R. T. Oh J. Nair G. Calabresi P. A. Davatzikos C. Doshi J. (2017). Volumetric analysis from a harmonized multisite brain MRI study of a single subject with multiple sclerosis. AJNR Am. J. Neuroradiol. 38 (8), 1501–1509. 10.3174/ajnr.a5254 28642263 PMC5557658

[B193] Silvoster M. L. Mathusoothana S. Kumar R. (2020). Efficient segmentation of lumbar intervertebral disc from MR images. IET Image Process. 14 (13), 3076–3083. 10.1049/iet-ipr.2019.0971

[B194] Sinaga K. P. Yang M.-S. (2020). Unsupervised K-means clustering algorithm. IEEE Access 8, 80716–80727. 10.1109/access.2020.2988796

[B195] Sneag D. B. Potter H. G. (2017). “3 imaging of the healthy and diseased spinal disc,” in Biological Approaches to Spinal Disc Repair and Regeneration for Clinicians, 20. 10.1055/b-0037-145105

[B196] Snoeckx A. Reyntiens P. Desbuquoit D. Spinhoven M. J. Van Schil P. E. van Meerbeeck J. P. (2018). Evaluation of the solitary pulmonary nodule: size matters, but do not ignore the power of morphology. Insights Imaging 9 (1), 73–86. 10.1007/s13244-017-0581-2 29143191 PMC5825309

[B197] Sozen T. Ozisik L. Basaran N. C. (2017). An overview and management of osteoporosis. Eur. J. Rheumatol. 4 (1), 46–56. 10.5152/eurjrheum.2016.048 28293453 PMC5335887

[B198] Stein T. Rau A. Russe M. F. Arnold P. Faby S. Ulzheimer S. (2023). Photon-counting computed tomography - basic principles, potenzial benefits, and initial clinical experience. ROFO 195 (8), 691–698. 10.1055/a-2018-3396 36863367

[B199] Stern D. Likar B. Pernus F. Vrtovec T. (2011). Parametric modelling and segmentation of vertebral bodies in 3D CT and MR spine images. Phys. Med. Biol. 56 (23), 7505–7522. 10.1088/0031-9155/56/23/011 22080628

[B200] Steyerberg E. W. Harrell F. E. Jr (2016). Prediction models need appropriate internal, internal-external, and external validation. J. Clin. Epidemiol. 69, 245–247. 10.1016/j.jclinepi.2015.04.005 25981519 PMC5578404

[B201] Stone M. (1974). Cross-validatory choice and assessment of statistical predictions. J. Royal Statistical Society Ser. B Methodol. 36 (2), 111–133. 10.1111/j.2517-6161.1974.tb00994.x

[B202] Tamez-Pena J. G. Totterman S. Parker K. J. (1999). “Unsupervised statistical segmentation of multispectral volumetric MRI images,” Medical Imaging 1999: Image Processing (Bellingham, WA: SPIE).

[B203] Tao R. Liu W. Zheng G. (2022). Spine-transformers: vertebra labeling and segmentation in arbitrary field-of-view spine CTs *via* 3D transformers. Med. Image Anal. 75, 102258. 10.1016/j.media.2021.102258 34670147

[B204] Tibshirani R. (1996). Regression shrinkage and selection *via* the lasso. J. R. Stat. Soc. Ser. B Methodol. 58 (1), 267–288. 10.1111/j.2517-6161.1996.tb02080.x

[B205] Tkachev S. (2024). The power of radiomics: machine learning predicts MRI features of resorption of the lumbar disc herniation. Spine J. 24 (9S), S141–S142. 10.1016/j.spinee.2024.06.076

[B64] U.S. Food and Drug Administration (2021). Good Machine Learning Practice for Medical Device Development: Guiding Principles. Silver Spring, MD: U.S. Food and Drug Administration.

[B206] U.S. Cancer Statistics Working Group, U.S. Cancer Statistics Data Visualizations Tool (2022). Cancer Facts & Figures 2022. Atlanta, GA: Centers for Disease Control and Prevention, U.S. Department of Health and Human Services.

[B207] Van den Brande R. Cornips E. M. Peeters M. Ost P. Billiet C. Van de Kelft E. (2022). Epidemiology of spinal metastases, metastatic epidural spinal cord compression and pathologic vertebral compression fractures in patients with solid tumors: a systematic review. J. Bone Oncol. 35, 100446. 10.1016/j.jbo.2022.100446 35860387 PMC9289863

[B208] van Griethuysen J. J. M. Fedorov A. Parmar C. Hosny A. Aucoin N. Narayan V. (2017). Computational radiomics system to decode the radiographic phenotype. Cancer Res. 77 (21), e104–e107. 10.1158/0008-5472.CAN-17-0339 29092951 PMC5672828

[B209] van Timmeren J. E. Cester D. Tanadini-Lang S. Alkadhi H. Baessler B. (2020). Radiomics in medical imaging-“how-to” guide and critical reflection. Insights Imaging 11 (1), 91. 10.1186/s13244-020-00887-2 32785796 PMC7423816

[B210] Ventura C. Cacioppa L. M. Caldarelli S. Sallei G. Lamponi F. Mascitti M. (2024). Toward the application of dual-energy computed tomography with virtual non-hydroxyapatite color-coded maps to identify traumatic fractures in daily emergency settings. J. Imaging 10 (11), 267. 10.3390/jimaging10110267 39590731 PMC11595750

[B211] Vo N. V. Hartman R. A. Patil P. R. Risbud M. V. Kletsas D. Iatridis J. C. (2016). Molecular mechanisms of biological aging in intervertebral discs. J. Orthop. Res. 34 (8), 1289–1306. 10.1002/jor.23195 26890203 PMC4988945

[B212] Waldenberg C. Brisby H. Hebelka H. Lagerstrand K. M. (2023). Associations between vertebral localized contrast changes and adjacent annular fissures in patients with low back pain: a radiomics approach. J. Clin. Med. 12 (15), 4891. 10.3390/jcm12154891 PMC10420134 37568293

[B213] Walker S. H. Duncan D. B. (1967). Estimation of the probability of an event as a function of several independent variables. Biometrika 54 (1), 167–179. 10.1093/biomet/54.1-2.167 6049533

[B214] Wallner J. Hochegger K. Chen X. Mischak I. Reinbacher K. Pau M. (2018). Clinical evaluation of semi-automatic open-source algorithmic software segmentation of the mandibular bone: practical feasibility and assessment of a new course of action. PLoS One 13 (5), e0196378. 10.1371/journal.pone.0196378 29746490 PMC5944980

[B215] Wang H. Hu D. Editors (2005). “Comparison of SVM and LS-SVM for Regression.” in 2005 International Conference on Neural Networks and Brain (IEEE).

[B216] Wang Y. X. J. Diacinti D. Yu W. Cheng X. G. Nogueira-Barbosa M. H. Che-Nordin N. (2020). Semi-quantitative grading and extended semi-quantitative grading for osteoporotic vertebral deformity: a radiographic image database for education and calibration. Ann. Transl. Med. 8 (6), 398. 10.21037/atm.2020.02.23 32355842 PMC7186643

[B217] Wang J. Zhou S. Chen S. He Y. Gao H. Yan L. (2023). Prediction of osteoporosis using radiomics analysis derived from single source dual energy CT. BMC Musculoskelet. Disord. 24 (1), 100. 10.1186/s12891-022-06096-w 36750927 PMC9903590

[B218] Wang M. Chen X. Cui W. Wang X. Hu N. Tang H. (2023). A computed tomography-based radiomics nomogram for predicting osteoporotic vertebral fractures: a longitudinal study. J. Clin. Endocrinol. Metab. 108 (6), e283–e294. 10.1210/clinem/dgac722 36494103

[B219] Wang X. Zhou D. Kong Y. Cheng N. Gao M. Zhang G. (2023). Value of (18)F-FDG-PET/CT radiomics combined with clinical variables in the differential diagnosis of malignant and benign vertebral compression fractures. EJNMMI Res. 13 (1), 89. 10.1186/s13550-023-01038-6 PMC10567613 37819414

[B220] Wang J. He Y. Yan L. Chen S. Zhang K. (2024). Predicting osteoporosis and osteopenia by fusing deep transfer learning features and classical radiomics features based on single-source dual-energy CT imaging. Acad. Radiol. 31 (10), 4159–4170. 10.1016/j.acra.2024.04.022 38693026

[B221] Wang S. Tong X. Fan Y. Hu M. Cui J. Li J. (2024). Combining deep learning and radiomics for automated, objective, comprehensive bone mineral density assessment from low-dose chest computed tomography. Acad. Radiol. 31 (3), 1180–1188. 10.1016/j.acra.2023.08.030 37730494

[B222] Wang L. Diacinti D. Diacinti D. Minisola S. Yajun L. (2025). The diagnosis of osteoporosis, including opportunistic computed tomography: a narrative review. Gerontology 71 (6), 453–460. 10.1159/000545961 40359912

[B249] Wang Z. Zhang H. Li Y. Zhang X. Liu J. Ren Z. (2026). Development and validation of a prognostic prediction model for lumbar-disc herniation based on machine learning and fusion of clinical text data and radiomic features. Eur. Spine J. 35 (2), 529–547. 10.1007/s00586-025-09102-6 40586925

[B223] Wasserthal J. Breit H. C. Meyer M. T. Pradella M. Hinck D. Sauter A. W. (2023). TotalSegmentator: robust segmentation of 104 anatomic structures in CT images. Radiol. Artif. Intell. 5 (5), e230024. 10.1148/ryai.230024 37795137 PMC10546353

[B224] Wewel J. T. O'Toole J. E. (2020). Epidemiology of spinal cord and column tumors. Neurooncol Pract. 7 (1), i5–i9. 10.1093/nop/npaa046 33299568 PMC7705531

[B225] Wichtmann B. D. Harder F. N. Weiss K. Schönberg S. O. Attenberger U. I. Alkadhi H. (2023). Influence of image processing on radiomic features from magnetic resonance imaging. Investig. Radiol. 58 (3), 199–208. 10.1097/rli.0000000000000921 36070524

[B226] Willemink M. J. Persson M. Pourmorteza A. Pelc N. J. Fleischmann D. (2018). Photon-counting CT: technical principles and clinical prospects. Radiology 289 (2), 293–312. 10.1148/radiol.2018172656 30179101

[B227] Wu W. Parmar C. Grossmann P. Quackenbush J. Lambin P. Bussink J. (2016). Exploratory study to identify radiomics classifiers for lung cancer histology. Front. Oncology 6, 71. 10.3389/fonc.2016.00071 27064691 PMC4811956

[B228] Wu S. Zhan W. Liu L. Xie D. Yao L. Yao H. (2023). Pretreatment radiomic biomarker for immunotherapy responder prediction in stage IB-IV NSCLC (LCDigital-IO study): a multicenter retrospective study. J. Immunother. Cancer 11 (10), e007369. 10.1136/jitc-2023-007369 37865396 PMC10603353

[B229] Xie Q. Chen Y. Hu Y. Zeng F. Wang P. Xu L. (2022). Development and validation of a machine learning-derived radiomics model for diagnosis of osteoporosis and osteopenia using quantitative computed tomography. BMC Med. Imaging 22 (1), 140. 10.1186/s12880-022-00868-5 PMC9358842 35941568

[B230] Xie J. Yang Y. Jiang Z. Zhang K. Zhang X. Lin Y. (2024). MRI radiomics-based decision support tool for a personalized classification of cervical disc degeneration: a two-center study. Front. Physiology 14, 1281506. 10.3389/fphys.2023.1281506 38235385 PMC10791783

[B231] Xue C. Yuan J. Lo G. G. Chang A. T. Y. Poon D. M. C. Wong O. L. (2021). Radiomics feature reliability assessed by intraclass correlation coefficient: a systematic review. Quant. Imaging Med. Surg. 11 (10), 4431–4460. 10.21037/qims-21-86 34603997 PMC8408801

[B232] Xue Z. Huo J. Sun X. Sun X. Ai S. T. Zhang L. (2022). Using radiomic features of lumbar spine CT images to differentiate osteoporosis from normal bone density. BMC Musculoskelet. Disord. 23 (1), 336. 10.1186/s12891-022-05309-6 35395769 PMC8991484

[B233] Yang H. Yan S. Li J. Zheng X. Yao Q. Duan S. (2022). Prediction of acute *versus* chronic osteoporotic vertebral fracture using radiomics-clinical model on CT. Eur. J. Radiol. 149, 110197. 10.1016/j.ejrad.2022.110197 35149339

[B234] Yang Y. Wang S. Stevens G. M. Fan J. Wang A. S. (2025). Optimal weighting strategies for maximizing contrast-to-noise ratio in photon counting CT images. Med. Phys. 52 (2), 750–770. 10.1002/mp.17489 39447021

[B235] Yang J. Zhang S. B. Yang S. Ge X. Y. Ren C. X. Wang S. J. (2025). CT-based radiomics predicts adjacent vertebral fracture after percutaneous vertebral augmentation. Eur. Spine J. 34 (2), 528–536. 10.1007/s00586-024-08579-x 39616589

[B236] Yao A. Sarkiss C. A. Ladner T. R. Jenkins A. L. 3rd (2017). Contemporary spinal oncology treatment paradigms and outcomes for metastatic tumors to the spine: a systematic review of breast, prostate, renal, and lung metastases. J. Clin. Neurosci. 41, 11–23. 10.1016/j.jocn.2017.04.004 28462790

[B237] Yoo H. J. (2025). Deep learning-based image reconstruction in musculoskeletal MRI. J. Korean Soc. Radiol. 86 (5), 567–586. 10.3348/jksr.2025.0015 41113364 PMC12531655

[B238] Zaworski C. Cheah J. Koff M. F. Breighner R. Lin B. Harrison J. (2021). MRI-Based texture analysis of trabecular bone for opportunistic screening of skeletal fragility. J. Clin. Endocrinol. Metab. 106 (8), 2233–2241. 10.1210/clinem/dgab342 33999148

[B239] Zeytinoglu M. Jain R. K. Vokes T. J. (2017). Vertebral fracture assessment: enhancing the diagnosis, prevention, and treatment of osteoporosis. Bone 104, 54–65. 10.1016/j.bone.2017.03.004 28285014

[B240] Zhang Y. Oikonomou A. Wong A. Haider M. A. Khalvati F. (2017). Radiomics-based prognosis analysis for non-small cell lung cancer. Sci. Reports 7 (1), 46349. 10.1038/srep46349 28418006 PMC5394465

[B241] Zhang X. Zhang Y. Zhang G. Qiu X. Tan W. Yin X. (2022). Deep learning with radiomics for disease diagnosis and treatment: challenges and potential. Front. Oncol. 12, 773840. 10.3389/fonc.2022.773840 35251962 PMC8891653

[B242] Zhang J. Liu J. Liang Z. Xia L. Zhang W. Xing Y. (2023). Differentiation of acute and chronic vertebral compression fractures using conventional CT based on deep transfer learning features and hand-crafted radiomics features. BMC Musculoskelet. Disord. 24 (1), 165. 10.1186/s12891-023-06281-5 36879285 PMC9987077

[B243] Zhang S. Liu M. Li S. Cui J. Zhang G. Wang X. (2023). An MRI-based radiomics nomogram for differentiating spinal metastases from multiple myeloma. Cancer Imaging 23 (1), 72. 10.1186/s40644-023-00585-4 37488622 PMC10367256

[B244] Zhang H. Yuan G. Wang C. Zhao H. Zhu K. Guo J. (2023). Differentiation of benign *versus* malignant indistinguishable vertebral compression fractures by different machine learning with MRI-based radiomic features. Eur. Radiol. 33 (7), 5069–5076. 10.1007/s00330-023-09678-x 37099176

[B245] Zhang B. Chen Z. Yan R. Lai B. Wu G. You J. (2024a). Development and validation of a feature-based broad-learning system for opportunistic osteoporosis screening using Lumbar spine radiographs. Acad. Radiol. 31 (1), 84–92. 10.1016/j.acra.2023.07.002 37495426

[B246] Zhang J. Xia L. Zhang X. Liu J. Tang J. Xia J. (2024b). Development and validation of a predictive model for vertebral fracture risk in osteoporosis patients. Eur. Spine J. 33 (8), 3242–3260. 10.1007/s00586-024-08235-4 38955868

[B247] Zhang J. Xia L. Liu J. Niu X. Tang J. Xia J. (2024c). Exploring deep learning radiomics for classifying osteoporotic vertebral fractures in X-ray images. Front. Endocrinol. (Lausanne) 15, 1370838. 10.3389/fendo.2024.1370838 PMC11007145 38606087

[B248] Zhang J. Xia L. Tang J. Xia J. Liu Y. Zhang W. (2024d). Constructing a deep learning radiomics model based on X-ray images and clinical data for predicting and distinguishing acute and chronic osteoporotic vertebral fractures: a multicenter study. Acad. Radiol. 31 (5), 2011–2026. 10.1016/j.acra.2023.10.061 38016821

[B250] Zhao Y. Zhao T. Chen S. Zhang X. Serrano Sosa M. Liu J. (2022). Fully automated radiomic screening pipeline for osteoporosis and abnormal bone density with a deep learning-based segmentation using a short lumbar mDixon sequence. Quant. Imaging Med. Surg. 12 (2), 1198–1213. 10.21037/qims-21-587 35111616 PMC8739149

[B251] Zhen T. Fang J. Hu D. Shen Q. Ruan M. (2024). Comparative evaluation of multiparametric lumbar MRI radiomic models for detecting osteoporosis. BMC Musculoskelet. Disord. 25 (1), 185. 10.1186/s12891-024-07309-0 38424582 PMC10902949

[B252] Zheng H. D. Sun Y. L. Kong D. W. Yin M. C. Chen J. Lin Y. P. (2022). Deep learning-based high-accuracy quantitation for lumbar intervertebral disc degeneration from MRI. Nat. Commun. 13 (1), 841. 10.1038/s41467-022-28387-5 35149684 PMC8837609

[B253] Zheng F. Yin P. Liang K. Liu T. Wang Y. Hao W. (2024). Comparison of different fusion radiomics for predicting benign and malignant sacral tumors: a pilot study. J. Imaging Inf. Med. 37 (5), 2415–2427. 10.1007/s10278-024-01134-6 38717515 PMC11522258

[B254] Zheng J. Liu W. Chen J. Sun Y. Chen C. Li J. (2024). Differential diagnostic value of radiomics models in benign *versus* malignant vertebral compression fractures: a systematic review and meta-analysis. Eur. J. Radiol. 178, 111621. 10.1016/j.ejrad.2024.111621 39018646

[B255] Zhong M. Liu J. T. Jiang H. (2017). Incidence of spontaneous resorption of lumbar disc herniation: a meta-analysis. Pain Physician 20 (1), E45–E52. 10.36076/ppj.2017.1.E45 28072796

[B256] Zhou C. Zhang G. Panchal R. R. Ren X. Xiang H. Xuexiao M. (2018). Unique complications of percutaneous endoscopic lumbar discectomy and percutaneous endoscopic interlaminar discectomy. Pain Physician 21 (2), E105–E112. 10.36076/ppj.2018.2.E105 29565953

[B257] Zhu W. S. Shi S. Y. Yang Z. H. Song C. Shen J. (2020). Radiomics model based on preoperative gadoxetic acid-enhanced MRI for predicting liver failure. World J. Gastroenterol. 26 (11), 1208–1220. 10.3748/wjg.v26.i11.1208 32231424 PMC7093309

[B258] Zhu H. N. Guo Y. F. Lin Y. Sun Z. C. Zhu X. Li Y. (2025). Radiomics analysis of thoracic vertebral bone marrow microenvironment changes before bone metastasis of breast cancer based on chest CT. J. Bone Oncol. 50, 100653. 10.1016/j.jbo.2024.100653 39712652 PMC11655691

[B259] Zhuang X. Wang J. Kang J. Lin Z. (2025). Diagnosis of acute *versus* chronic thoracolumbar vertebral compression fractures using CT radiomics based on machine learning: a preliminary study. J. Imaging Inf. Med. 38 (4), 2183–2193. 10.1007/s10278-024-01359-5 PMC12343428 39653874

[B260] Zou H. Hastie T. (2005). Regularization and variable selection via the elastic net. J. R. Stat. Soc. Ser. B Stat. Methodol. 67 (2), 301–320. 10.1111/j.1467-9868.2005.00503.x

[B261] Zwanenburg A. Vallieres M. Abdalah M. A. Aerts H. Andrearczyk V. Apte A. (2020). The image biomarker standardization initiative: standardized quantitative radiomics for high-throughput image-based phenotyping. Radiology 295 (2), 328–338. 10.1148/radiol.2020191145 32154773 PMC7193906

